# End-to-end multimodal pathology foundation model with clinical dialogue

**DOI:** 10.1038/s41591-026-04521-4

**Published:** 2026-07-31

**Authors:** Eugene Vorontsov, George Shaikovski, Adam Casson, Julian Viret, Eric Zimmermann, Neil Tenenholtz, Yi Kan Wang, Jan H. Bernhard, Ran A. Godrich, Juan A. Retamero, Jinru Shia, Mithat Gonen, Martin R. Weiser, David S. Klimstra, Razik Yousfi, Nicolò Fusi, Thomas J. Fuchs, Kristen Severson, Siqi Liu

**Affiliations:** 1 Paige, New York, NY USA; 2 Tempus, Chicago, IL USA; 3 Microsoft Research, Cambridge, MA USA; 4 Memorial Sloan Kettering Cancer Center, New York, NY USA; 5 Yale University, New Haven, CT USA

**Keywords:** Pathology, Cancer imaging, Cancer screening, Diagnostic markers, Prognostic markers

## Abstract

Recent rapid progress in the field of computational pathology has been enabled by foundation models. These models are beginning to move beyond encoding image patches toward whole-slide understanding, but their clinical utility remains limited. Here we present PRISM2, a multimodal slide-level foundation model trained on 2.3 million whole-slide images and 14 million question−answer pairs derived from 700,000 pathology reports. Through clinical dialogue supervision, PRISM2 aligns histomorphology with diagnostic reasoning, yielding representations that support both prompt-based inference and transferable embeddings for downstream tasks. With prompt-based inference, PRISM2 achieves or exceeds (*P* < 0.05) the balanced accuracy of clinical-grade products calibrated for cancer detection in the prostate, breast and breast lymph node. Additionally, across comprehensive diagnostic, biomarker and survival benchmarks, PRISM2 embeddings never statistically underperform previous foundation models via linear probing (*P* < 0.05). Furthermore, task-specific fine-tuning on survival prediction outperforms training from scratch on the same large survival dataset. PRISM2 demonstrates how language-supervised pretraining provides a scalable, clinically grounded signal for generalizable pathology representations, bridging human diagnostic reasoning and foundation model performance.

## Abstract (web summary)

Trained on 2.3 million whole-slide images and 14 million clinical question−answer pairs, the multimodal foundation model PRISM2 matched clinical-grade cancer detection performance without task-specific training, demonstrating the value of clinical dialogue supervision for computational pathology.

## Main

The field of computational pathology has been transformed by the development of foundation models. Models such as Virchow2 (ref. ^1^), UNI2 (ref. ^2^) and H-Optimus-1 (ref. ^3^) are trained using millions of histopathology tiles spanning diverse tissue types and employing self-supervised objectives^4^ to learn generalizable representations. These models improved performance, robustness and data efficiency in cancer detection, subtyping and biomarker quantification^2,5–8^. However, they remain fundamentally limited for case-level inference. Most high-value predictive tasks occur at the patient case level, which typically consists of one or more gigapixel whole-slide images (WSIs), and require aggregation of thousands of tiles. Current workflows predominantly train a task-specific tile aggregator using weakly supervised learning; however, this requires extensive data curation to avoid overfitting and poor robustness. Our work goes beyond this paradigm with, to our knowledge, the first evidence of a generalist model that can achieve the performance of specialized models authorized for clinical use without additional training.

**Fig. 1:**
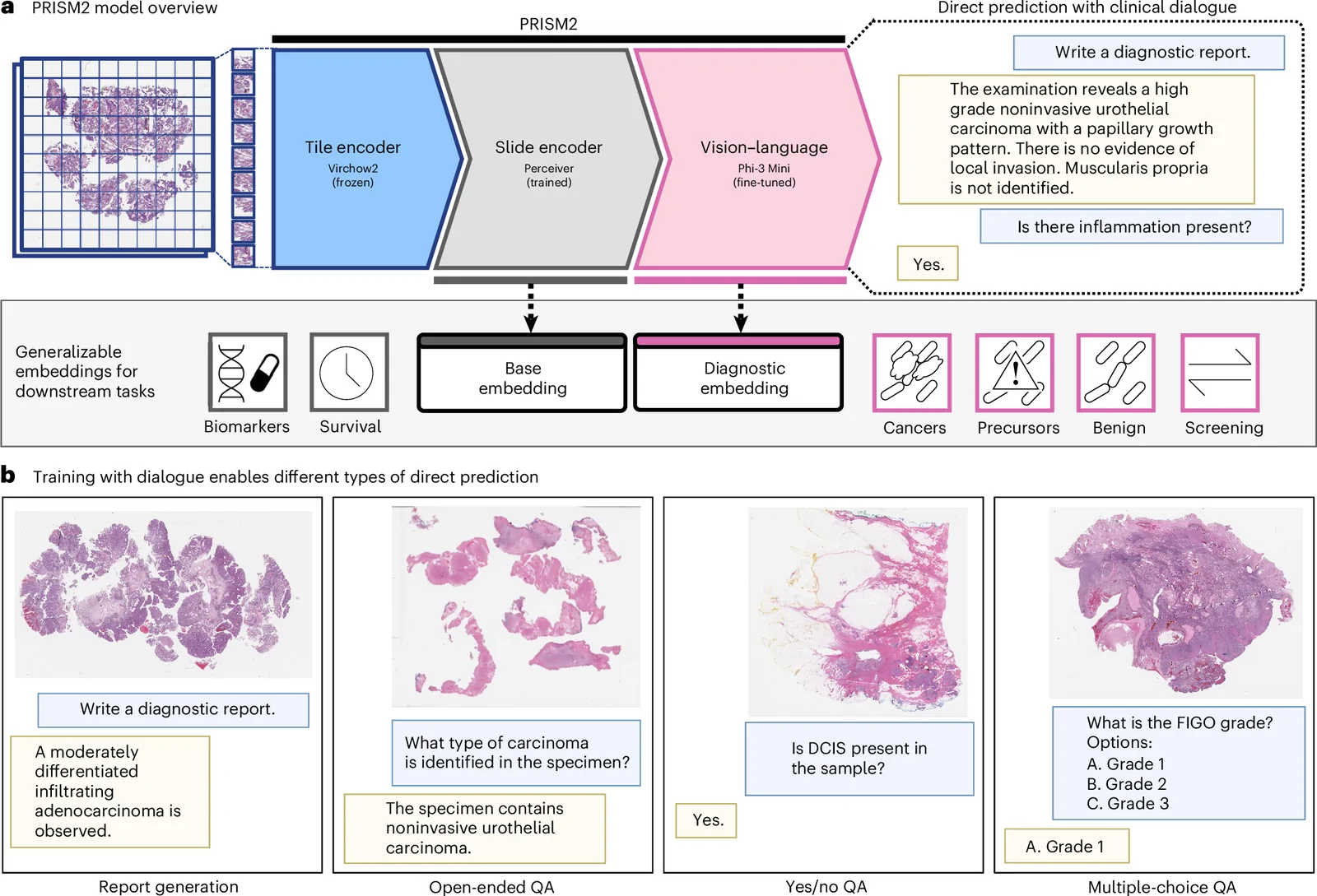
Overview of PRISM2. PRISM2 is a slide-level foundation model capable of producing generalizable embeddings as well as prompt-based inference with clinical dialogue. **a**, The input to PRISM2 is one or more WSIs associated with a patient case. These large tissue images are cropped into tiles, which are subsequently processed with the Virchow2 foundation model. The sequence of tile embeddings is then aggregated via a slide encoder into a base image representation (‘Base Embedding’). The base embedding is then used as input, along with text, to a large vision−language model that produces a diagnostic representation (‘Diagnostic Embedding’) and text. The diagnostic embedding is tailored to diagnostic tasks, such as the detection and identification of cancers, precursors to cancers and benign conditions. Base embeddings are more general and are well suited for transfer learning to tasks outside of the diagnosis-focused training distribution, such as biomarker and survival prediction. **b**, Examples of the four types of direct dialogue-based prediction templates: report generation, open-ended question answering, yes/no question answering and multiple-choice question answering. Yes/no and multiple-choice question answering in particular enable directly querying the model for quantifiable predictions with probabilities that can be calibrated. FIGO, Federation Internationale de Gynecologie et dʼObstetrique; QA, question answering.

**Fig. 2:**
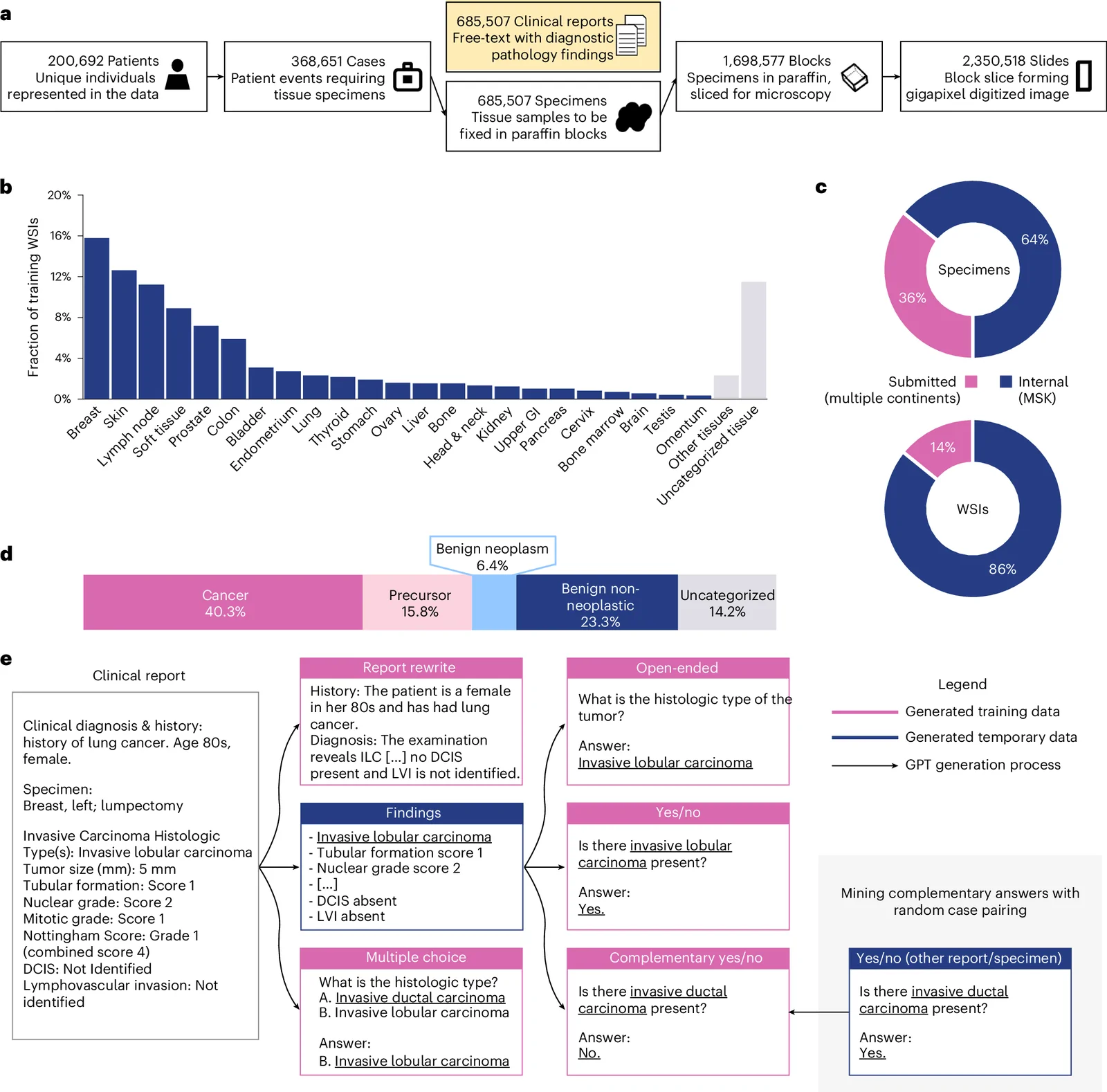
Overview of PRISM2 training data. **a**, The training data can be described in terms of patients, cases, specimens, blocks and slides, as shown. All WSIs are formalin-fixed, paraffin-embedded and stained with H&E. Clinical reports are paired at the level of specimens. Consequently, PRISM2 is trained at the specimen level to predict diagnostic findings in the clinical reports. **b**, The distribution of tissues represented in the training data. **c**, The distribution of specimens and WSIs between MSK samples and those submitted for a second opinion from diverse external sources around the world. **d**, Distribution of high-level conditions in the training data. **e**, Overview of how dialogue examples are generated from clinical reports to be used during PRISM2 training. Every processing step (arrow) uses GPT-4o. Clinical report rewrites are split into clinical history and pathology diagnosis. Diagnostic summaries are produced without demographic, molecular and sectioning information. Diagnostic findings are first listed and then converted into yes/no and open-ended question−answer pairs. As the yes/no question distribution is biased toward the findings mentioned in clinical reports, we mine additional complementary yes/no question−answer pairs by assuming that findings that are not mentioned are negative in the specimen. Multiple-choice question answering is derived directly from the reports, following CAP reporting guidelines. GI, gastrointestinal; LVI, lymphovascular invasion.

**Extended Data Fig. 1:**
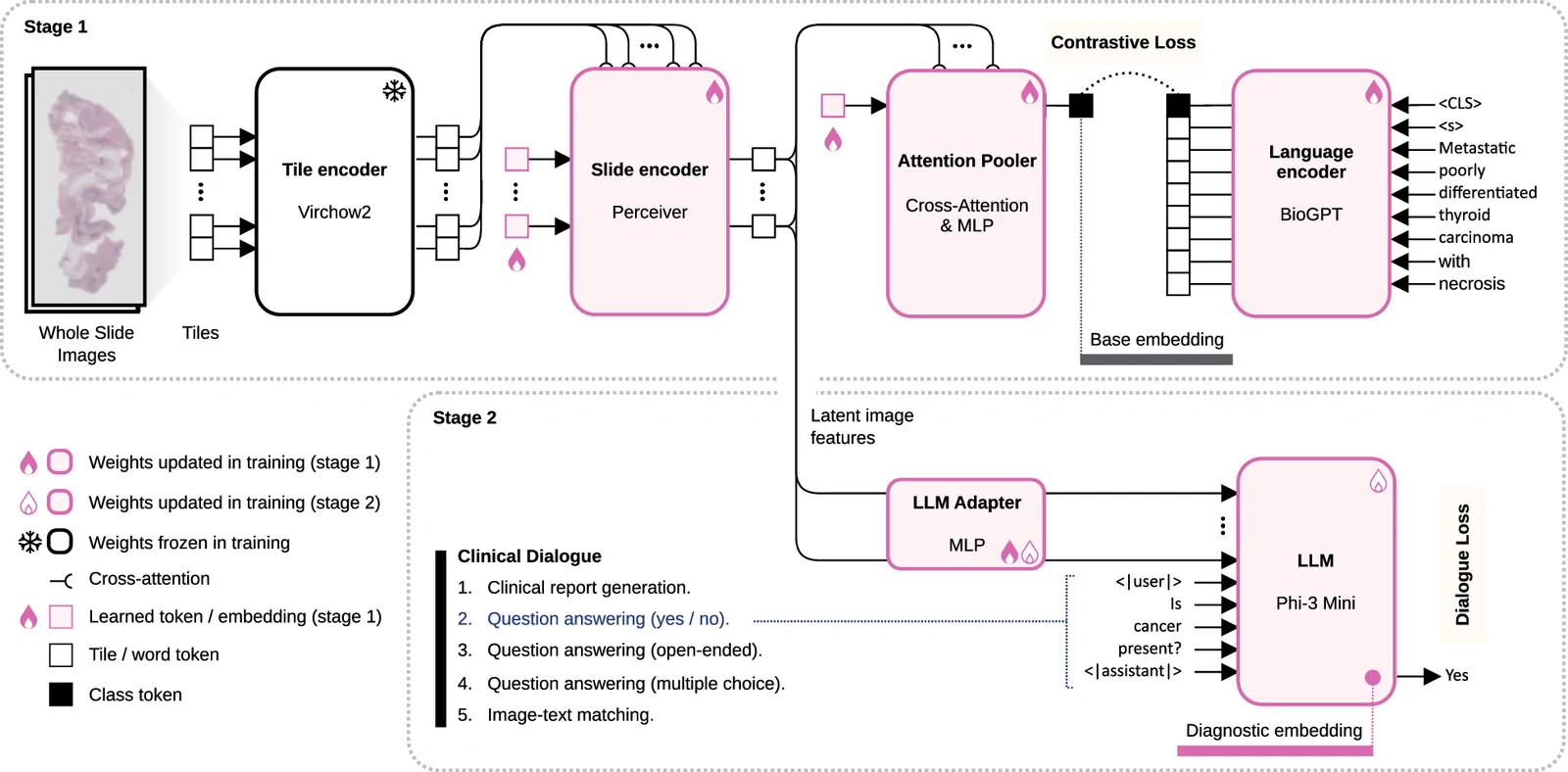
The architecture and training schematic for PRISM2. Sets of whole slide images are summarized as a base embedding (Attention Pooler class token) which is aligned to the representation of the clinical report diagnostic summary (encoded by the language decoder, BioGPT) by minimizing a contrastive loss. The image latent features are also passed to a pretrained large language model (LLM, Phi-3 Mini) along with a prompt from one of four dialogue templates, with the model’s response updated by minimizing the dialogue loss. Training proceeds in two stages: in stage 1, only the slide encoder, attention pooler, language decoder, and LLM adapter weights are updated; in stage 2, only the LLM adapter and LLM weights are updated. For use in downstream tasks, the latent image features produced by the slide encoder are summarized in two embeddings: (1) the features are pooled into a ‘base embedding’, used for contrastive alignment; and (2) the features are refined by the LLM into a ‘diagnostic embedding’, taken from the LLM hidden state at the <∣assistant∣> token, after inputting the image.

Here we present PRISM2 (Fig. 1 and Extended Data Fig. 1), a slide-level foundation model capable of clinical-grade predictive performance. A key challenge in creating slide-level foundation models is choosing a supervisory signal that is useful for compressing long sequences of tile representations while still being sufficiently generalizable to downstream tasks. Work to date has considered image-only^9,10^, paired molecular^11–13^ and clinical reports^14–17^ as sources for such signal (see Supplementary Table 1 for a summary). As scaling is well known to impact model performance^18–20^, PRISM2 leverages the scale and diversity enabled by learning from abundantly available paired clinical reports. Again to our knowledge, it is the largest multimodal slide-level pathology foundation model to date and is trained on the largest corpus of data, comprising 685,000 distinct clinical reports, corresponding to 2.3 million WSIs originating from diverse institutions and encompassing different tissue types (Fig. 2a−d and Methods). Unlike PRISM^15^ and TITAN^16^, which are slide-level foundation models also trained with report text, PRISM2 features a novel dual-embedding architecture: a lightweight ‘base’ embedding that transfers well to novel and complex tasks such as biomarker prediction and a ‘diagnostic’ embedding derived from the hidden state of a 4-billion-parameter language model, tuned for clinical tasks such as cancer detection and subtyping. Although PRISM2 is trained to replicate clinical dialogue, the goal is not to deploy it as a conversational agent but, rather, to use single-turn dialogue as a rich supervisory signal for learning semantically grounded and generalizable slide-level representations.

**Extended Data Fig. 2:**
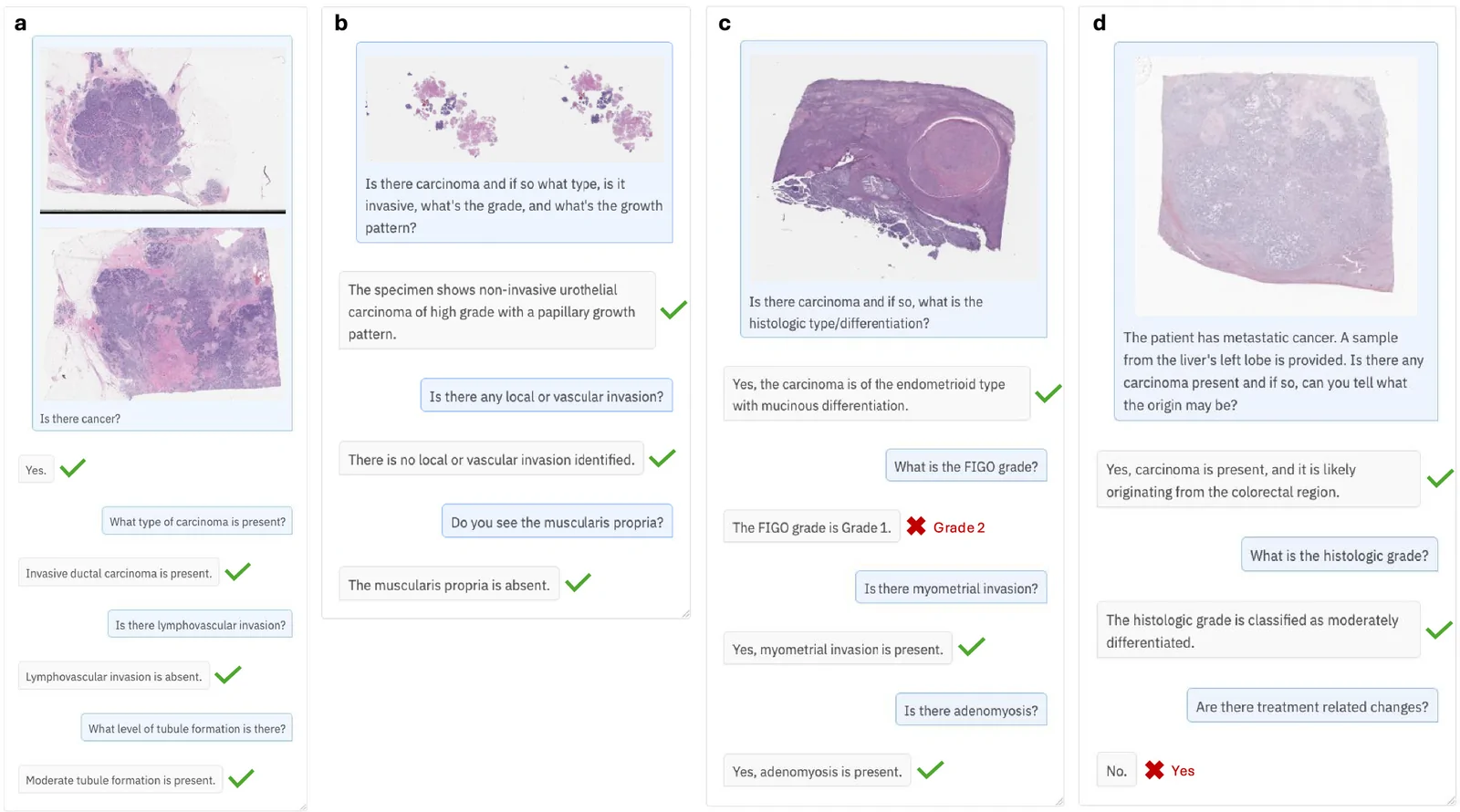
Examples of PRISM2 dialogues. **a** A breast specimen with IDC. We show WSIs in the initial message for illustrative purposes, but for each example PRISM2 views all the slides of the specimen at once. **b** A bladder biopsy. Despite being trained on simple questions, the model is able to accurately respond to compound questions. **c** A uterus specimen. The model correctly identifies and characterizes the carcinoma and additional findings except it incorrectly predicts FIGO Grade 1 instead of Grade 2. **d** A liver specimen from a patient with metastatic colorectal cancer. The model is able to be given additional context like some patient history and specimen description and detects the carcinoma and correctly predicts its origin and grade, but incorrectly says there are no treatment related changes.

PRISM2 is trained using a two-stage approach (Methods). In the first stage, a perceiver-based^21^ slide encoder aggregates Virchow2 (ref. ^1^) tile embeddings. This slide-level representation is aligned with diagnostic summary text using dual objectives: a contrastive objective using BioGPT^22^ text embeddings and an autoregressive objective using the Phi-3 Mini^20^ language model. Although inspired by the CoCa^15,23^ approach (also the algorithmic basis of PRISM and TITAN), PRISM2 is clearly differentiated via its use of clinical dialogue (Extended Data Fig. 2). Building upon insights from large language model (LLM) literature^20^, generating pathology report text alone is likely insufficient^24,25^. There is heterogeneity in pathology reporting protocols, and these are not always followed; information density can vary accordingly. To overcome this, PRISM2 introduces detailed question answering alongside report generation, a capability that is refined in the second stage of training where the slide encoder is frozen and the Phi-3 Mini language model is fine-tuned. This directly leverages the reasoning capabilities of modern LLMs, yielding a new question-answering capability that we refer to as ‘prompt-based inference’^26^.

Within a cancer-oriented pathology workflow, prompt-based inference enables PRISM2 to serve as a specialist agent for an interactive assistant model. Such an assistant could answer queries such as ’Is lymphovascular invasion present?’ during routine sign-out, provide a second opinion for general pathologists in remote areas or act as a digital resident to pre-populate fields in College of American Pathologists (CAP) synoptic worksheets. Furthermore, clinical products trained on PRISM2 embeddings could automatically prioritize and route high-risk cases to subspecialists, order confirmatory immunohistochemistry (IHC) or sequencing panels before pathologist review or predict primary tumor sites for metastases of unknown origin to guide stain orders.

To quantify the performance of PRISM2, we evaluated a suite of cancer diagnostic classification, biomarker prediction and survival tasks to capture the breadth of slide-level foundation model applications using several modeling approaches. Prompt-based inference without further training is performed using question answering. Linear probe evaluation measures how well the embeddings generalize to in-distribution and out-of-distribution tasks. Overall, we show improved performance by using language capabilities while maintaining state-of-the-art performance in applications not typically represented in clinical reports, such as biomarkers. Notably, without further training, PRISM2 achieves clinical-grade cancer detection when evaluated against clinical algorithms for prostate, breast and breast lymph node (BLN). Finally, we demonstrate, to our knowledge, the first application of a slide-level foundation model for clinical report completion, following CAP reporting guidelines. Taken together, these results demonstrate the value of large-scale, multimodal training.

## Results

We evaluate PRISM2 across multiple settings, as outlined below. Crucially, all evaluation data are unseen during PRISM2 and Virchow2 training.

### Clinical dialogue training achieves diagnostic-grade performance without task-specific training

Whereas tile-level computational pathology foundation models are trained with self-supervision on a pretext task, PRISM2, like other slide-level computational pathology foundation models, is trained to generate clinically relevant text. Consequently, for certain tasks, the model can achieve high performance without further training, a setting we call ‘prompt-based inference’^26^. We differentiate this from ‘zero-shot’ prediction, as the indications of interest (for example, invasive ductal or lobular carcinoma) are observed during training. PRISM2 performs prompt-based inference via its question-answering capabilities. To date, prompt-based inference in computational pathology has focused on the contrastive setting (for example, refs. ^15,16^), where candidate labels are compared with image embeddings and the nearest label in embedding space is selected. We evaluate the prompt-based inference of PRISM2 using question answering and PRISM and TITAN using contrastive comparison (see ‘Evaluation methods’ for evaluation protocols, ‘Comparison models’ for comparison models and ‘Evaluation datasets’ for evaluation datasets).

**Fig. 3:**
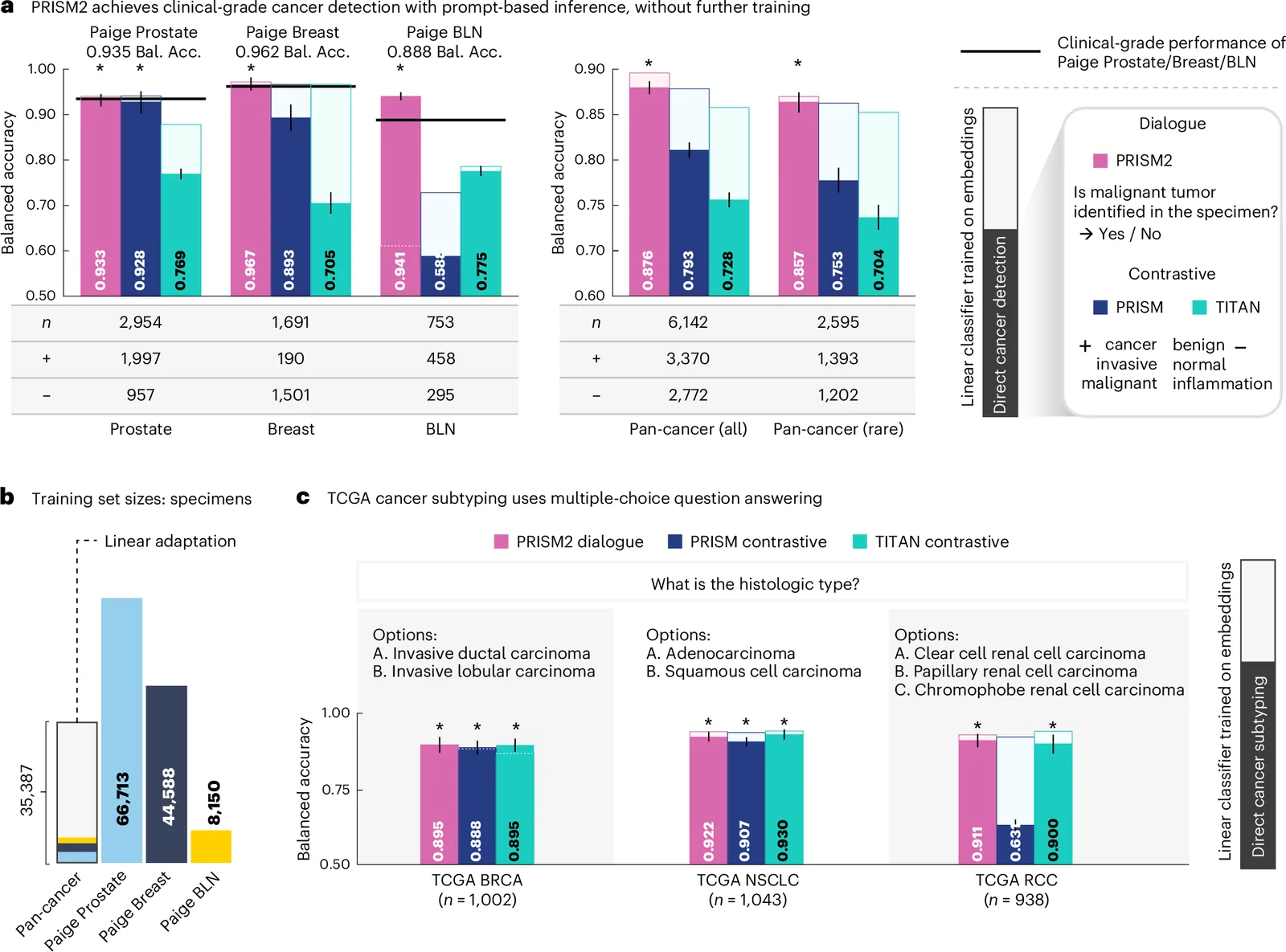
Prompt-based inference through dialogue with PRISM2. **a**, Yes/no question answering matches or exceeds the performance of existing clinical-grade products for the detection of invasive cancer as compared to Paige Prostate, Paige Breast and Paige BLN, when evaluated on the corresponding product testing datasets. Yes/no question answering on pan-cancer and rare variants exceeds the contrastive performance of competing approaches. After training a linear classifier using the diagnostic embedding as input on a large pan-cancer dataset, PRISM2 pan-cancer performance improves and continues to outperform competing approaches with similar adaptation. **b**, The size of the pan-cancer training set used for linear probing is similar to the training sets of the three clinical models; however, prostate (light blue), breast (dark blue) and BLN (yellow) tissues are a relatively small subset of the pan-cancer dataset. **c**, Prompt-based inference performs well on TCGA cancer subtyping with multiple-choice question answering. In all panels, *n* indicates the number of samples; + and − indicate the number of positive and negative samples, respectively. Error bars show the 95% confidence interval computed with bootstrapping. * indicates the best prompt-based inference result as well as any models tied for first place as measured by a two-sided unadjusted permutation test with *P* < 0.05.

First, we demonstrate prompt-based inference performance on tissue-specific cancer detection (Fig. 3a), comparing with specialist commercial models: Paige Prostate, Paige Breast and Paige BLN. These models were developed with rigorous clinical validation and large, curated datasets, as summarized in Fig. 3b. Paige Prostate has FDA De Novo approval for clinical use in the United States. Paige BLN has an FDA Breakthrough Device designation. All three products are CE-IVD and UKCA marked for clinical diagnostic use in Europe and the UK. Using yes/no question answering (Supplementary Table 3), PRISM2 matches the performance of Paige Prostate and Paige Breast and outperforms Paige BLN without further training (Fig. 3a). Neither PRISM nor TITAN match the performance of the clinical-grade products with contrastive classification, showing a particularly notable performance gap on BLN. PRISM and TITAN improve with linear probing (shaded region) but still trail PRISM2.

For pan-cancer prediction, PRISM2 performs best overall (Fig. 3a). Although PRISM2 prompt-based inference is similar to linear pan-cancer prediction on prostate, breast and BLN product testing datasets, linear probing improves results on the pan-cancer test set with nine common and seven rare cancer types by tumor origin tissue. This suggests that, for some cancer types, training with PRISM2 diagnostic embeddings can improve performance over prompt-based inference. Indeed, for all three models, linear probing improves performance. Nevertheless, as with prompt-based inference, only PRISM2 matches or outperforms all three clinical-grade products with linear probing.

We also evaluate prompt-based subtyping on The Cancer Genome Atlas (TCGA) data^27,28^ for breast, lung and kidney cancers (Fig. 3c; see Supplementary Table 4 for multiple-choice question prompts). Models perform similarly, except PRISM in the kidney task.

**Extended Data Fig. 3:**
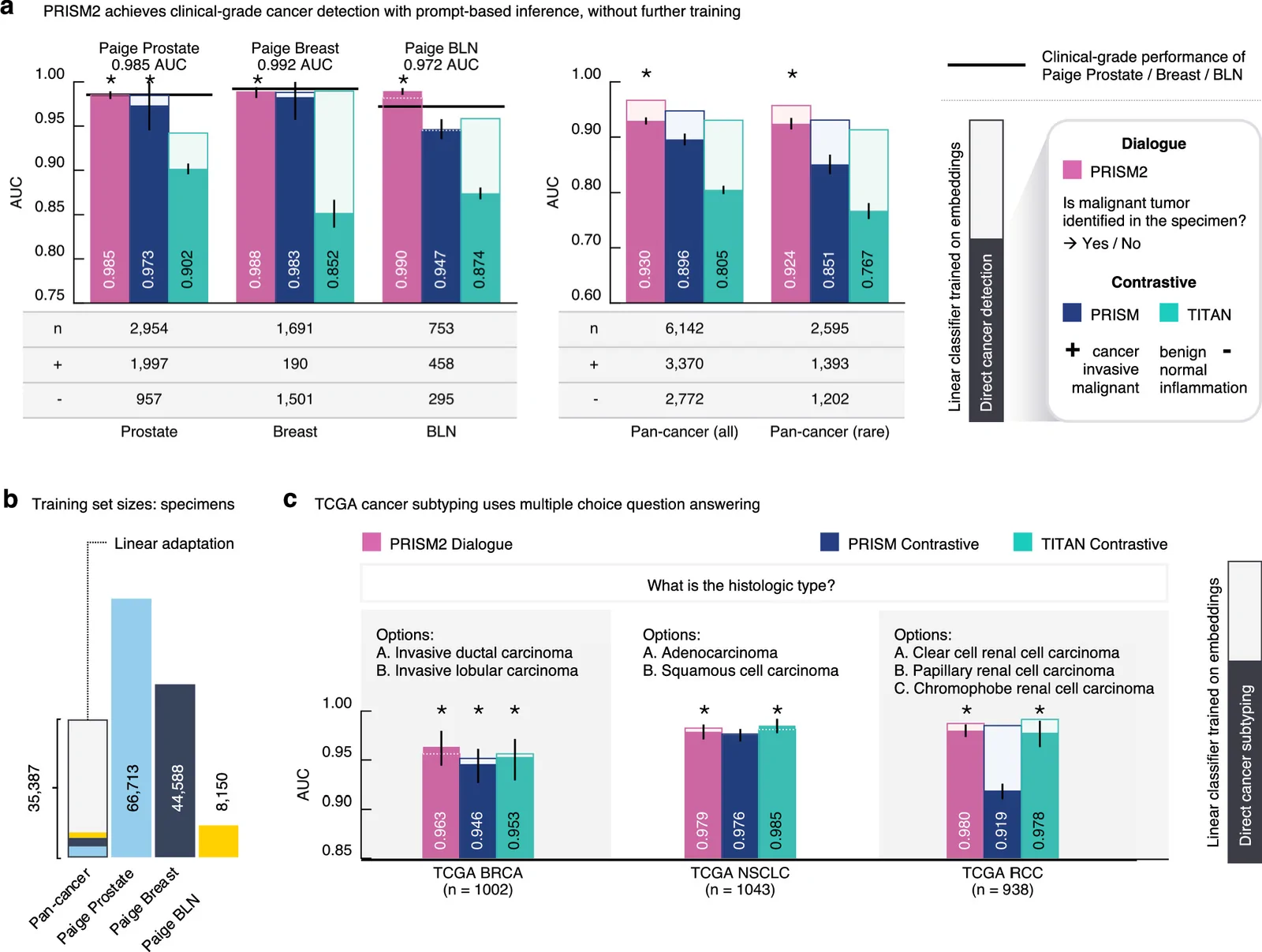
Prompt-based inference through dialogue with PRISM2 (AUC). **a** Yes-no QA matches or exceeds clinical-grade AI product performance for the detection of invasive cancer as compared to Paige Prostate, Paige Breast, and Paige Breast Lymph Node (BLN), when evaluated on the corresponding product testing datasets. When training a linear classifier on a large pan-cancer dataset, prompt-based inference by PRISM2 is as good as the linear classifier on the product testing datasets (Prostate, Breast, BLN) and nearly as good as the linear classifier on the pan-cancer dataset, including the rare cancers subset. **b** The size of the pan-cancer training set used for linear probing is similar to the training sets of the three clinical models; however, prostate (light blue), breast (dark blue), and BLN (yellow) tissues are a relatively small subset of the pan-cancer dataset. **c** Prompt-based inference performs well on TCGA cancer subtyping with multiple-choice QA. In all panels, *n* indicates number of samples; + and − indicate number of positive and negative samples, respectively. Error bars show the 95% confidence interval computed with bootstrapping. * prompt-based inference result tied for first place (p < 0.05, two-sided unadjusted permutation test).

To calculate balanced accuracy (Fig. 3a), prompt-based inference and linear probing use a probability threshold of 0.5, whereas clinical-grade products use carefully tuned thresholds. However, threshold-free area under the curve (AUC) (Extended Data Fig. 3a) yields the same conclusion: PRISM2 prompt-based inference matches or outperforms clinical-grade products in cancer detection, but no threshold allows either PRISM or TITAN to do the same, even with linear probing on a large pan-cancer dataset.

### PRISM2 diagnostic embeddings are well suited to diagnostic pathology tasks

**Extended Data Fig. 4:**
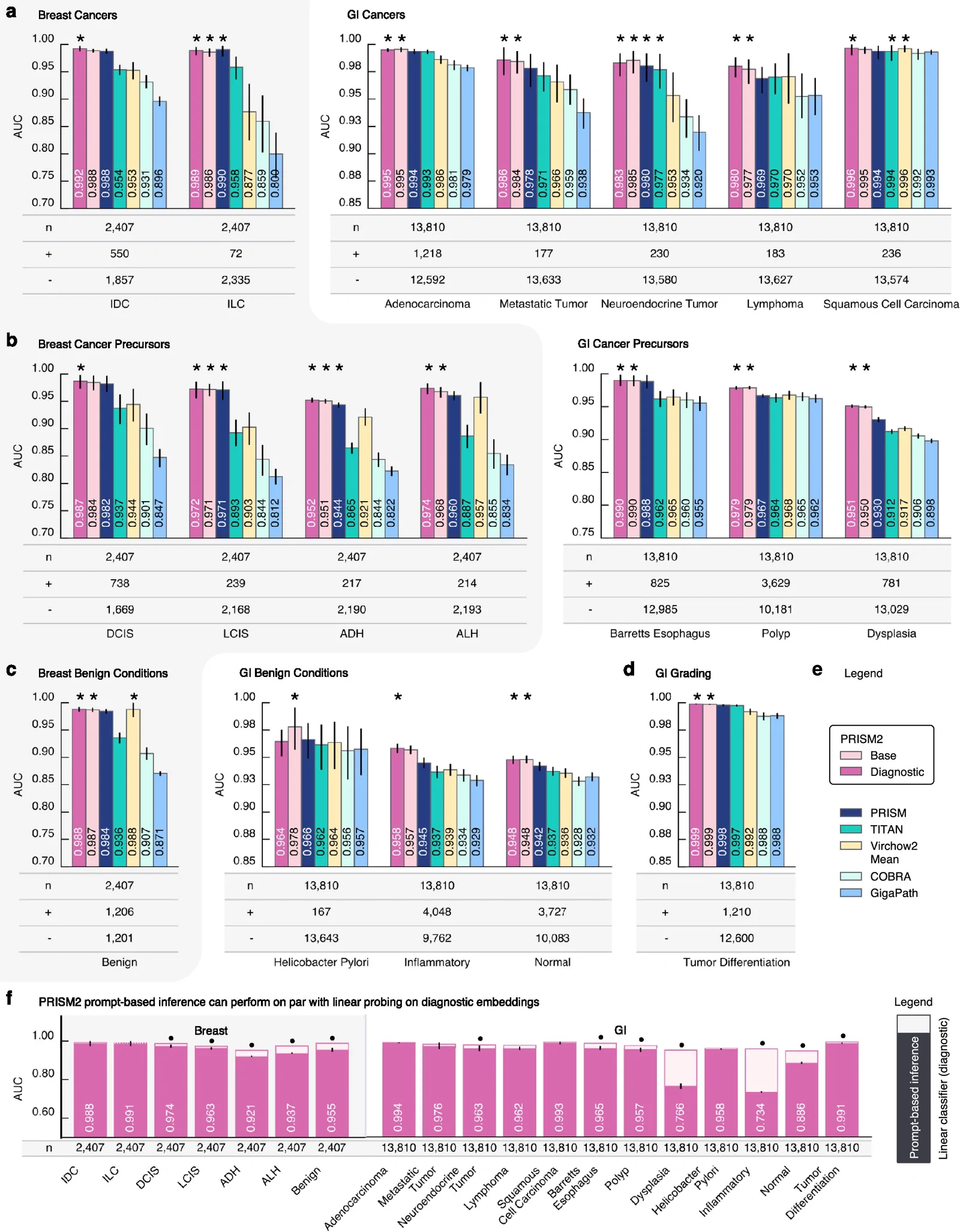
Adapting slide-level model embeddings to downstream diagnostic pathology tasks via linear probing. The detection of breast and GI cancers. Panels **a**-**d** show the AUC performance for the detection of cancers (**a**), precursors to cancer (**b**), and benign conditions (**c**) for breast and gastrointestinal (GI) tissues, as well as cancer grading for GI (**d**); legend in **e**. **f** Yes-no QA enables prompt-based detection without further training at or near the performance of the linear classifier, especially for cancers. In all panels, *n* indicates number of samples; + and − indicate number of positive and negative samples, respectively. Error bars show 95% confidence intervals computed with bootstrapping. * linear probe result tied for first place (p < 0.05, permutation test); • prompt-based inference differs from linear probe result (p < 0.05, permutation test).

As noted above, due to model architecture and training design choices, PRISM2 generates two types of embeddings: base and diagnostic. As is standard for foundation models, we expect these embeddings to generalize to many downstream tasks. To evaluate these representations, we gathered datasets to assess the ability of different models to identify cancers, precursors and benign conditions. Specifically, we gathered pan-cancer, pan-cancer (rare), breast and gastrointestinal detection tasks from Memorial Sloan Kettering Cancer Center (MSK), subtyping tasks for breast, lung and kidney cancer from TCGA^27,28^ and CAMELYON16 (refs. ^29,30^), CAMELYON17 (ref. ^31^), PANDA^32^ and IMP-CRS^33–35^ from additional external public data sources. The large MSK datasets present challenging real-world pathology tasks. The pan-cancer task involves detection of invasive cancer across 16 cancer tissues of origin (seven comprising the ‘rare’ cancers subset). The breast and gastrointestinal tasks involve detecting cancers, precursors to cancer and benign conditions (see Extended Data Fig. 4 for per-condition results). Tasks using data external to MSK assess important clinical questions. TCGA tasks involve cancer subtyping in breast, lung and kidney. CAMELYON16 and CAMELYON17 involve tumor detection and staging, respectively, in lymph node resections. PANDA and IMP-CRS involve prostate and colorectal tumor grading, respectively. Dataset details are in ‘Evaluation datasets’.

To compare slide-level embeddings, we evaluate PRISM^15^, TITAN^16^, COBRA^36^ and Prov-GigaPath^37^. Additionally, we evaluate the mean of the Virchow2 (ref. ^1^) tile embeddings, representing a naive aggregation baseline. In all cases, the slide embedding is input to a linear model. See ‘Evaluation methods’ and ‘Comparison models’ for methodology and model details, respectively.

**Fig. 4:**
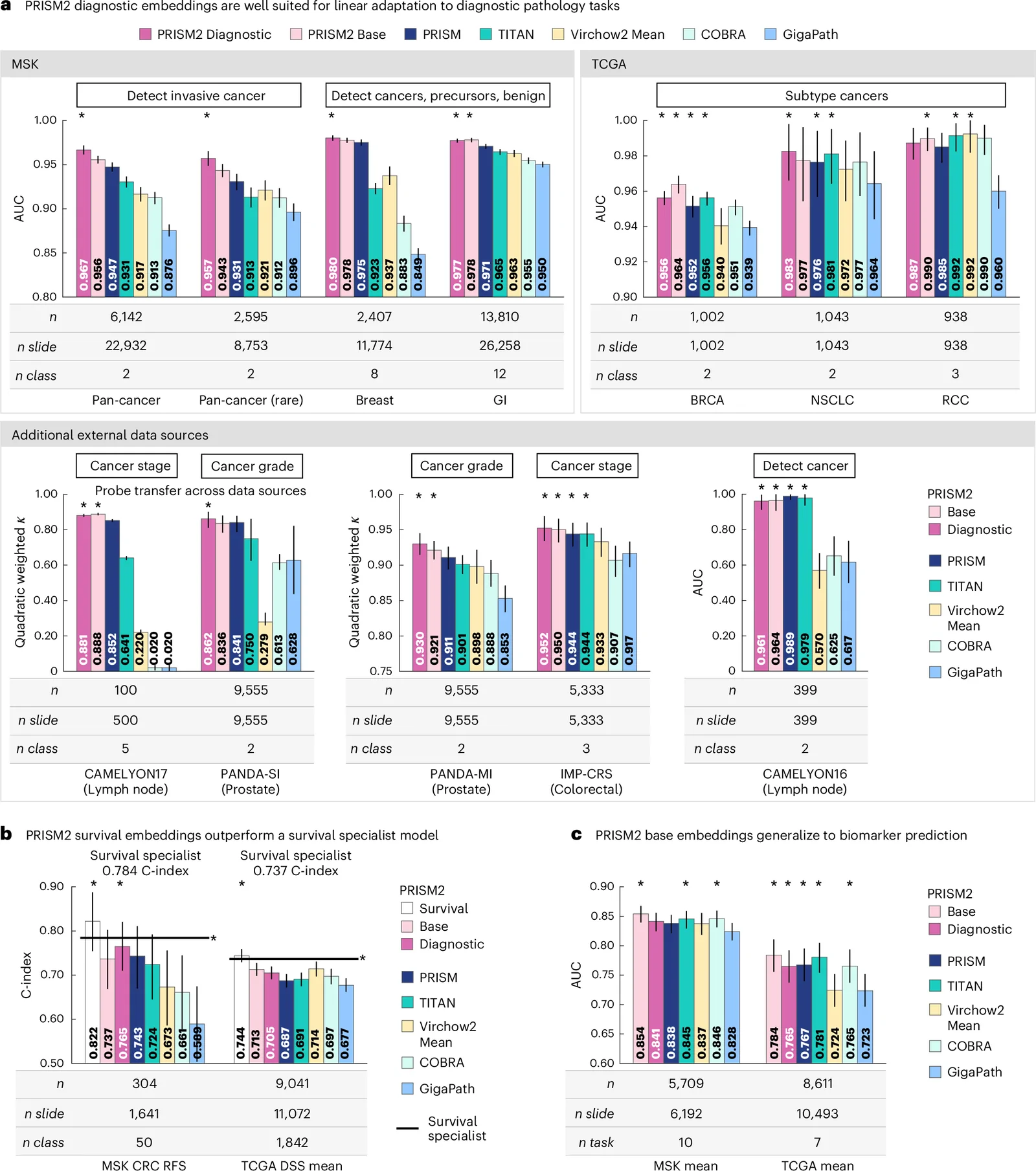
Adapting slide-level model embeddings to downstream tasks. **a**, Linear probing with PRISM2 diagnostic embeddings outperforms all other embeddings on a set of challenging tasks, including pan-cancer detection and the detection and identification (typing) of cancers, precursors to cancer and benign conditions in breast and gastrointestinal tissues, as well as cancer subtyping, staging, grading in lymph node and prostate using external public data. **b**,**c**, PRISM2 base embeddings are better suited for generalization beyond diagnostic tasks. **b**, Survival task performance evaluated with Cox regression for CRC RFS with MSK data and DSS averaged over 13 tasks with TCGA data. PRISM2 survival embeddings outperform all others and match or exceed the performance of the survival specialist model, followed by PRISM2 base. **c**, PRISM2 base embeddings perform at least as well as those of any other model for biomarker detection, as evaluated via linear probing. In all panels, *n* indicates the number of samples; *n slide*, *n class*, *n event* and *n task* indicate the number of WSIs, classes, recurrence or death events and tasks, respectively. Error bars show the 95% confidence interval computed with bootstrapping. * indicates the best linear probe result as well as any models tied for first place as measured by a two-sided unadjusted permutation test with *P* < 0.05.

Figure 4a presents performance on standard clinical tasks. In all settings, PRISM2 base and diagnostic embeddings match or outperform others. For pan-cancer detection, PRISM2 diagnostic embeddings (0.967 AUC) substantially outperform both PRISM2 base (0.956 AUC) and all other embeddings (0.947 AUC with PRISM, 0.931 AUC with TITAN). Furthermore, the performance drop on rare cancers is modest (0.967 to 0.957 AUC). In breast, PRISM2 and PRISM embeddings outperform others. PRISM2 embeddings yield the highest linear probe performance in gastrointestinal across all cancer, precursor and benign indications (Extended Data Fig. 4) with TITAN competitive on cancer, with no significant difference in two of six labels (neuroendocrine and squamous cell carcinoma), but falling behind on precursors and benign conditions. Although the COBRA slide-level model uses Virchow2 tile embeddings, its embeddings often underperform the baseline method of taking a mean of the Virchow2 tile embeddings, suggesting the importance of the slide-level training stage.

Experiments using external data further support the strength of PRISM2 embeddings. Of note, CAMELYON17 tumor staging yielded the most variable performance, and only three models—PRISM2, PRISM and TITAN—achieved quadratic weighted *κ* scores greater than 0.5, with PRISM2 diagnostic and base (0.881 and 0.888) significantly (*P* < 0.05) outperforming PRISM (0.852) and TITAN (0.641). Institution-specific confounders are unlikely as evaluation involves five-fold cross-validation across source institutions, requiring cross-institution linear probe transfer. This transfer is also necessary for prostate cancer grading with PANDA-SI, where PRISM2 diagnostic embeddings significantly (*P* < 0.05) outperformed all other embeddings, achieving 0.862 quadratic weighted (compared to 0.836 with base, 0.841 with PRISM and 0.750 with TITAN). PRISM2 diagnostic and base achieved the highest score (0.930 and 0.921) when institutions were mixed in PANDA-MI, narrowing the gap to baselines (0.911 and 0.901 for PRISM and TITAN, respectively). Finally, PRISM2 diagnostic and base, alongside PRISM and TITAN, achieved the top scores (*P* < 0.05) in IMP-CRS colorectal cancer (CRC) grading and CAMELYON16 lymph node cancer detection.

### PRISM2 survival embeddings outperform a survival specialist model

Accurate prognostic prediction is a major focus of computational pathology but remains challenging due to data scarcity and uncertain connections between morphology and patient outcome^38,39^. We assess the benefit of pretraining with report data, and of additional fine-tuning, for survival prediction tasks. We gathered a dataset of more than 225,000 cases tracking overall survival across nearly 100,000 patients (Fig. 4c). We fine-tuned the PRISM2 slide encoder and trained a survival specialist model from scratch (‘PRISM2 training’). We refer to the fine-tuned model’s embeddings as ‘survival’ embeddings. We evaluated on CRC recurrence-free survival (RFS) prediction with MSK data and 13 disease-specific survival (DSS) prediction tasks with TCGA data (‘Evaluation datasets’).

**Extended Data Fig. 5:**
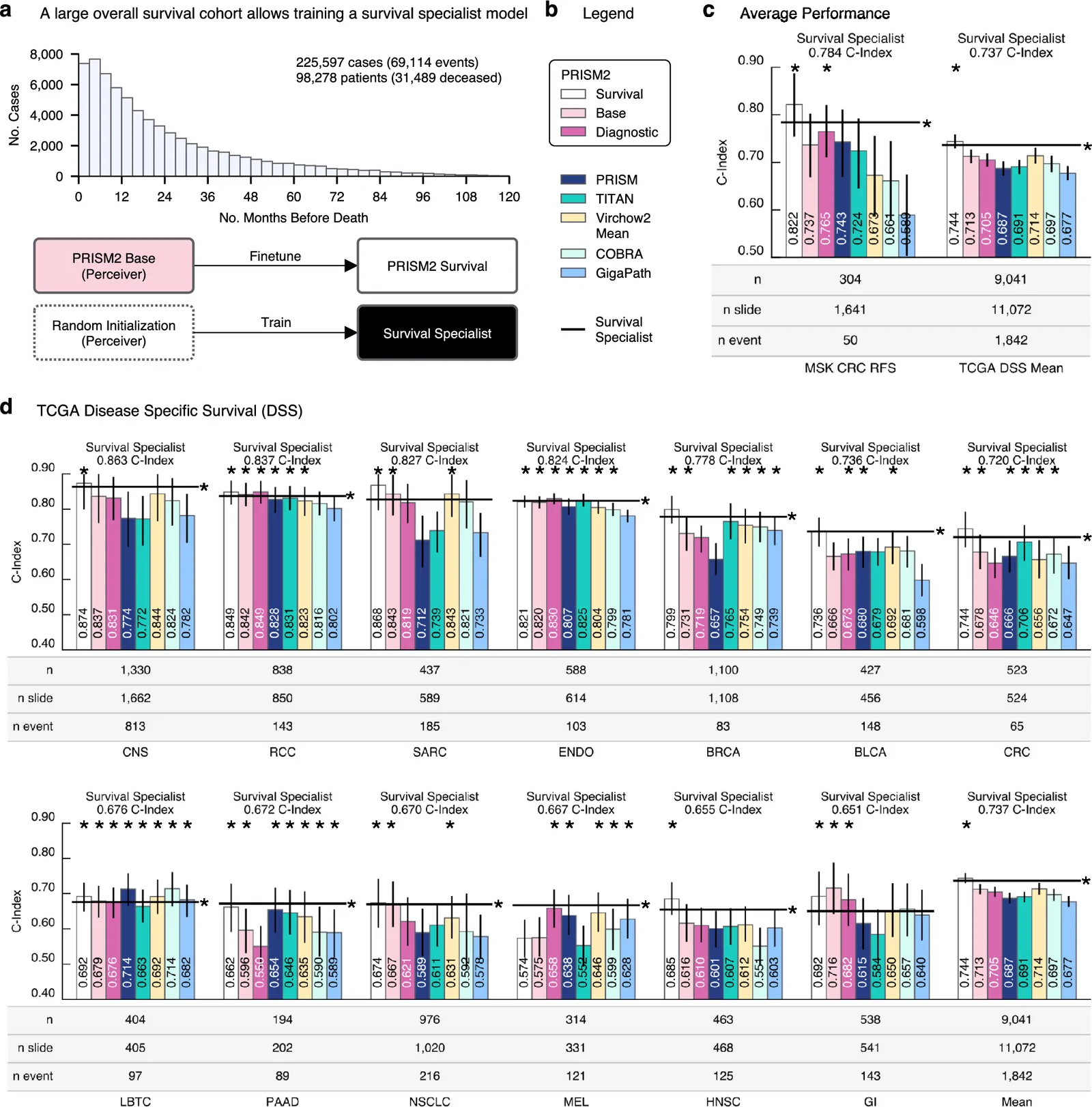
Adapting slide-level model embeddings to survival prediction. **a** A large overall survival (OS) cohort was used to train the survival specialist model as well as the PRISM2 Survival model, by fine-tuning the slide encoder (base embedding). **b**-**d** Survival task performance evaluated with linear Cox regression (C-Index) for Colorectal cancer (CRC) recurrence-free survival (RFS) with MSK data and disease-specific survival (DSS) for TCGA data (legend in **b**). **c** PRISM2 Survival embeddings outperform all others and match or exceed the performance of the survival specialist model, followed by PRISM2 Base. **d** Per-task DSS prediction on TCGA data. In all panels, *n* indicates number of samples; *n slide* and *n event* indicate number of WSIs and recurrence or death events, respectively. Error bars show 95% confidence intervals computed with bootstrapping. * survival result tied for first place (p < 0.05, two-sided unadjusted permutation test).

PRISM2 survival embeddings outperform the survival specialist model (Fig. 4b), especially for MSK CRC RFS (0.809 versus 0.773 concordance index (C-index)). Without survival fine-tuning, PRISM2 base embeddings outperform all other embeddings for MSK CRC RFS and match the naive Virchow2 mean baseline on TCGA DSS. Unsurprisingly, base embeddings slightly outperform diagnostic embeddings, as survival information was absent in the dialogue training data (see Extended Data Fig. 5 for per-task results).

### PRISM2 base embeddings generalize to biomarker prediction

**Extended Data Fig. 6:**
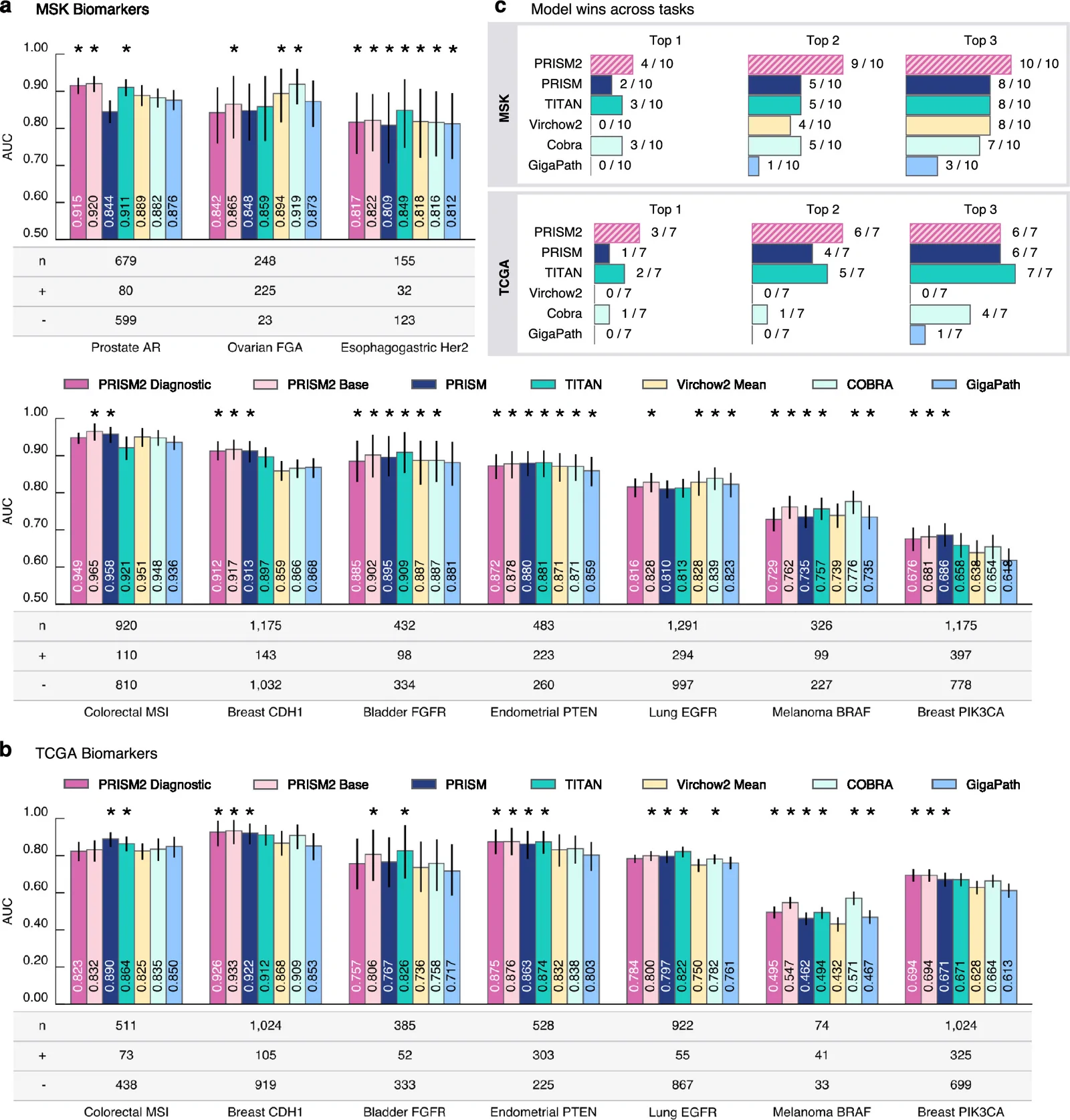
Adapting slide-level model embeddings to biomarker prediction. Linear probing on model embeddings, evaluated with AUC. **a** MSK biomarker prediction tasks. **b** TCGA biomarker prediction tasks. **c** The number of times each model’s embeddings score within the top 1, 2, and 3 best-performing embeddings across tasks, as measured by the AUC rounded to within two decimal points. In all panels, *n* indicates number of samples; + and − indicate number of positive and negative samples, respectively. Error bars show 95% confidence intervals computed with bootstrapping. * linear classifier result tied for first place (p < 0.05, two-sided unadjusted permutation test).

Biomarker prediction using hematoxylin and eosin (H&E) WSIs could extend the applications of computational pathology, with early results showing great promise^7,8^. We selected 10 tissue-specific biomarker datasets useful for treatment decisions and evaluated if PRISM2 embeddings can replace typical genetic sequencing. To assess generalizability beyond MSK data, we selected the seven biomarkers from TCGA that overlap with our curated subset (‘Evaluation datasets’). Fig. 4d,e and Extended Data Fig. 6 present the results. Similar to survival prediction, PRISM2 base embeddings outperform diagnostic embeddings. Although average AUCs on MSK TCGA tasks are highest for PRISM2 base embeddings, the next-best-performing models are close: 0.846 (versus 0.854) AUC with COBRA on MSK and 0.781 (versus 0.784) AUC on TCGA with TITAN. PRISM2 embeddings rank more often among the top 1, top 2 and top 3 per biomarker prediction task by a small margin (Extended Data Fig. 4c). Overall, PRISM2 embeddings perform at least as well as other models for biomarker prediction.

### Dialogue unlocks pathology report completion

As a final demonstration of prompt-based inference with dialogue, we move beyond benchmark to evaluate the ability of PRISM2 to complete routine pathology reports, following CAP guidelines. To our knowledge, we are the first to explore detailed report completion directly with a pathology foundation model without further training, illustrating its potential in clinical practice.

**Fig. 5:**
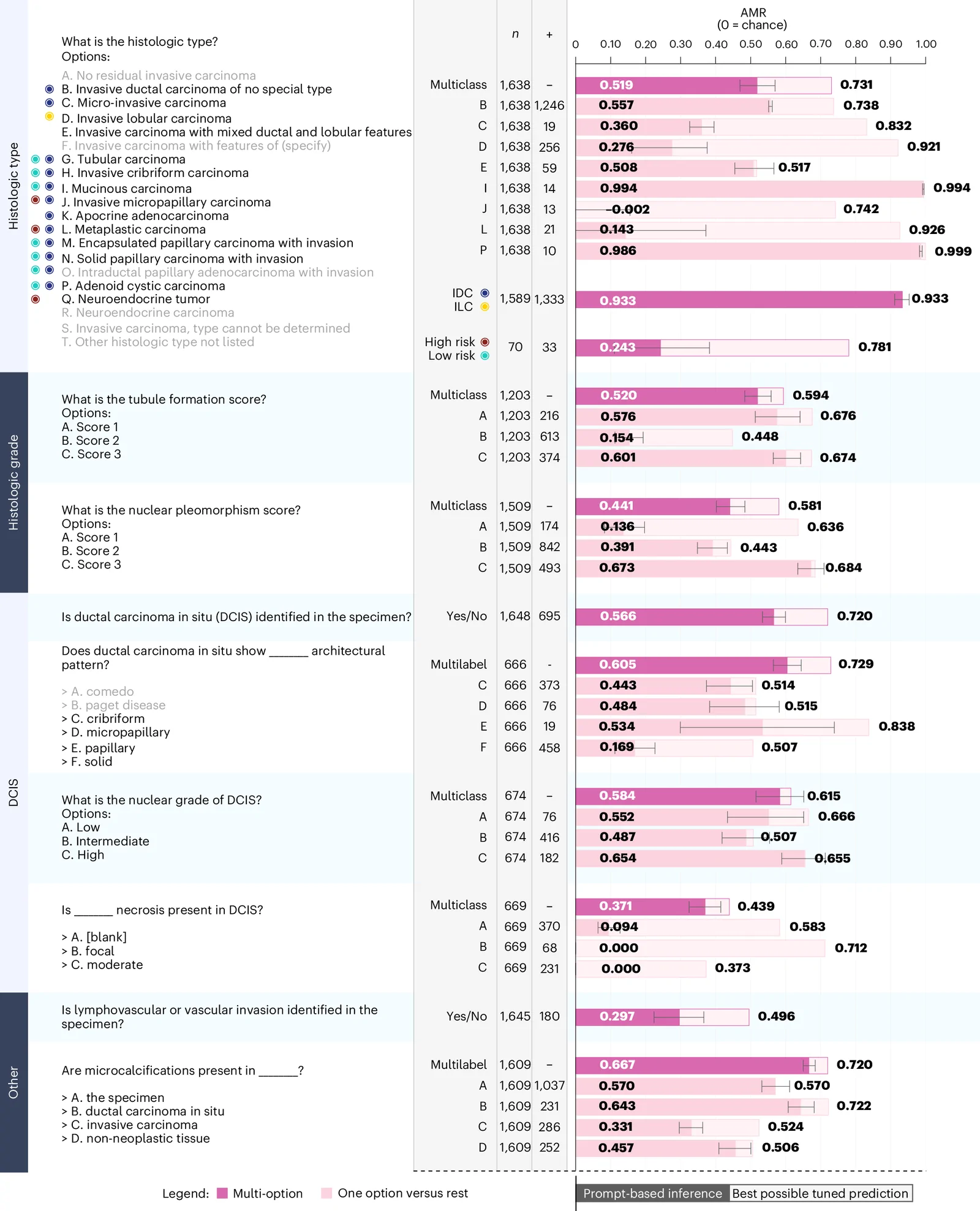
Direct completion of pathology filling is enabled with prompt-based inference. Multiple-choice and yes/no question answering allows for directly filling a clinical report following CAP guidelines, demonstrated here for invasive carcinoma of the breast (biopsy). Left: questions posed to PRISM2. For multiple-choice questions (marked ‘Multiclass’), all options are provided to the model at the same time. For yes/no questions (marked ‘Yes/No’ (binary) or ‘Multilabel’), each option in a multilabel report field completes the blank in the yes/no question. Right: predictive performance is measured with mean recall across all classes, adjusted such that randomly assigned values score 0 (for example, 0.5 is adjusted to 0 for binary; 0.25 is adjusted to 0 for four classes). AMR is computed only across options with at least 10 examples in the evaluation data. Included options are shown with black text and excluded options are shown with gray text. AMR of the PRISM2 prompt-based inference is shown (number in bar) as a fraction of the maximum performance achievable by tuning per-class probability thresholds or weights (number right of bar). For histologic type, the gap between these is minimal when considering IDC (blue dot) versus ILC (yellow dot), but large when considering high-risk (red dot) versus low-risk (cyan dot) types, suggesting that the relevant information is present in the model, but its prediction is poorly calibrated for these classes. *n* indicates the number of samples, and + indicates the number of positive samples. Error bars show the 95% confidence interval computed with bootstrapping.

We demonstrate this capability with the CAP report template for biopsies with invasive carcinoma of the breast^40^ (methods in ‘Evaluation methods’ and dataset in ‘Evaluation datasets’). Figure 5 shows report field questions and performance metrics. The fully shaded bars show the adjusted mean recall (AMR) from question answering (‘observed AMR’), and the lightly shaded extensions to these bars show the maximum AMR by calibrating response probabilities (‘max AMR’). Histologic type, ductal carcinoma in situ (DCIS) presence, DCIS architectural pattern and lymphovascular invasion fields benefit substantially from calibration. Tubule formation score, DCIS nuclear grade, necrosis and microcalcifications benefit less. Overall, PRISM2 shows modest to good understanding of the diagnostic concepts, but some responses require calibration.

Selecting the correct histologic type among 20 options is challenging. Only 14 options were represented in the evaluation data, with eight (B, C, D, E, I, J, L, P) having at least 10 samples (‘*n*’ and ‘+’ in Fig. 5). PRISM2 achieved an AMR of 0.519 (0.731 with calibration) across these options. Mucinous carcinoma (I) and adenoid cystic carcinoma (P) are the only options where observed AMR (0.994) matches max AMR (0.986); however, the AMR for invasive ductal carcinoma (IDC) of no special type (B) and invasive lobular carcinoma (ILC) (D) is low (0.557 and 0.276). TCGA breast cancer results in Fig. 3c and breast IDC and ILC results in Extended Data Fig. 4f show that the model identifies these indications well. Identification becomes difficult when including all six ductal carcinoma variants (B, C, I, J, L, P) and the ductal and lobular mixed type (E). Indeed, a stratified analysis of histologic type classification reveals that IDC versus ILC performance is good (0.933 AMR) and does not benefit from calibration. Conversely, pooling rare histologic subtypes as prognostic of high risk or low risk requires calibration.

### Slide-level diagnostic descriptions are consistent with highly attended regions

**Extended Data Fig. 7:**
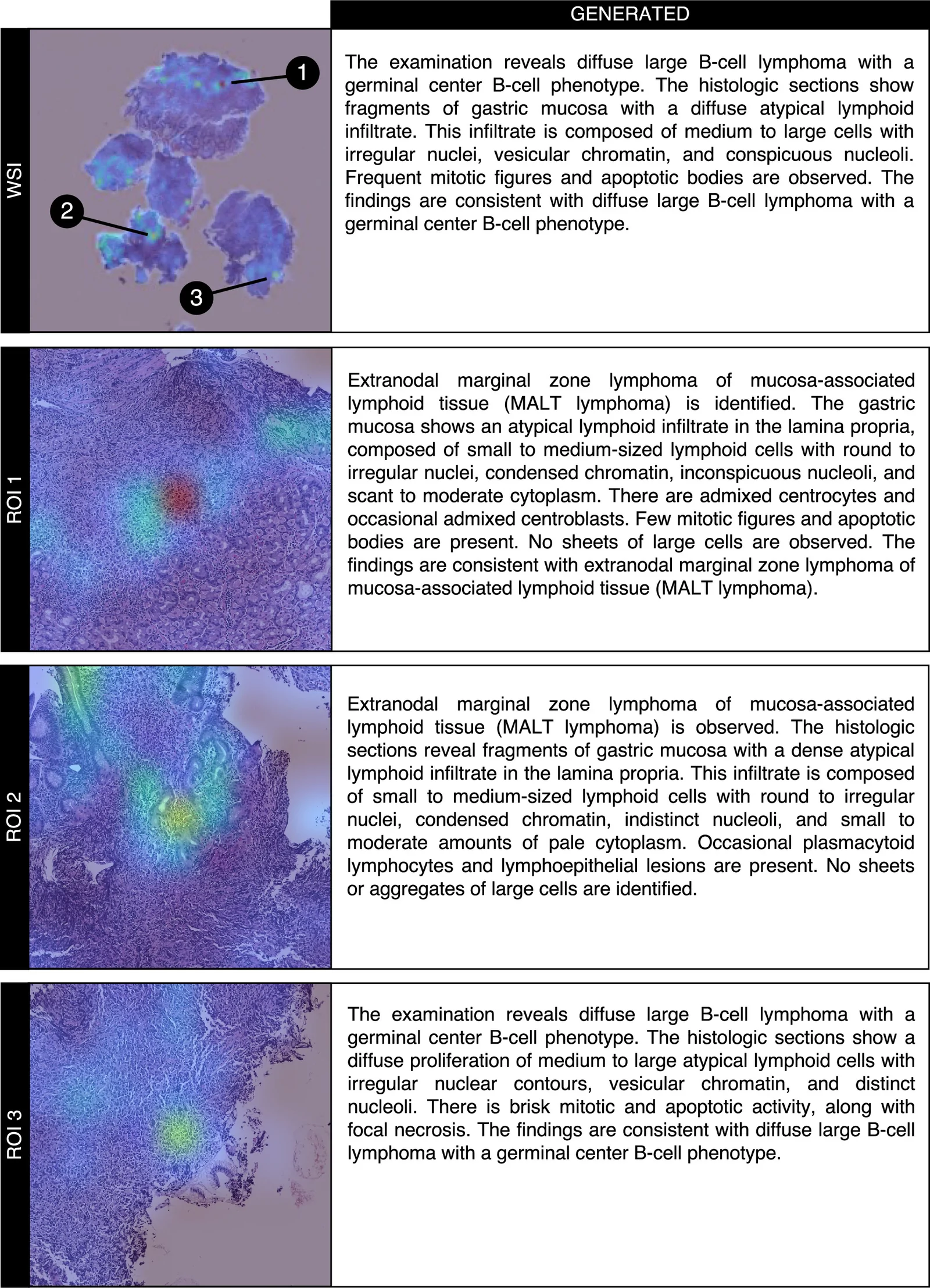
Lymphoma sample. The slide encoder attention heatmap is shown for a core in a single slide of a lymphoma specimen, along with the diagnostic report generated by PRISM2. When cropping to each of the three top-attended regions, the PRISM2 report remains consistent with the evidence in the regions of interest.

**Extended Data Fig. 8:**
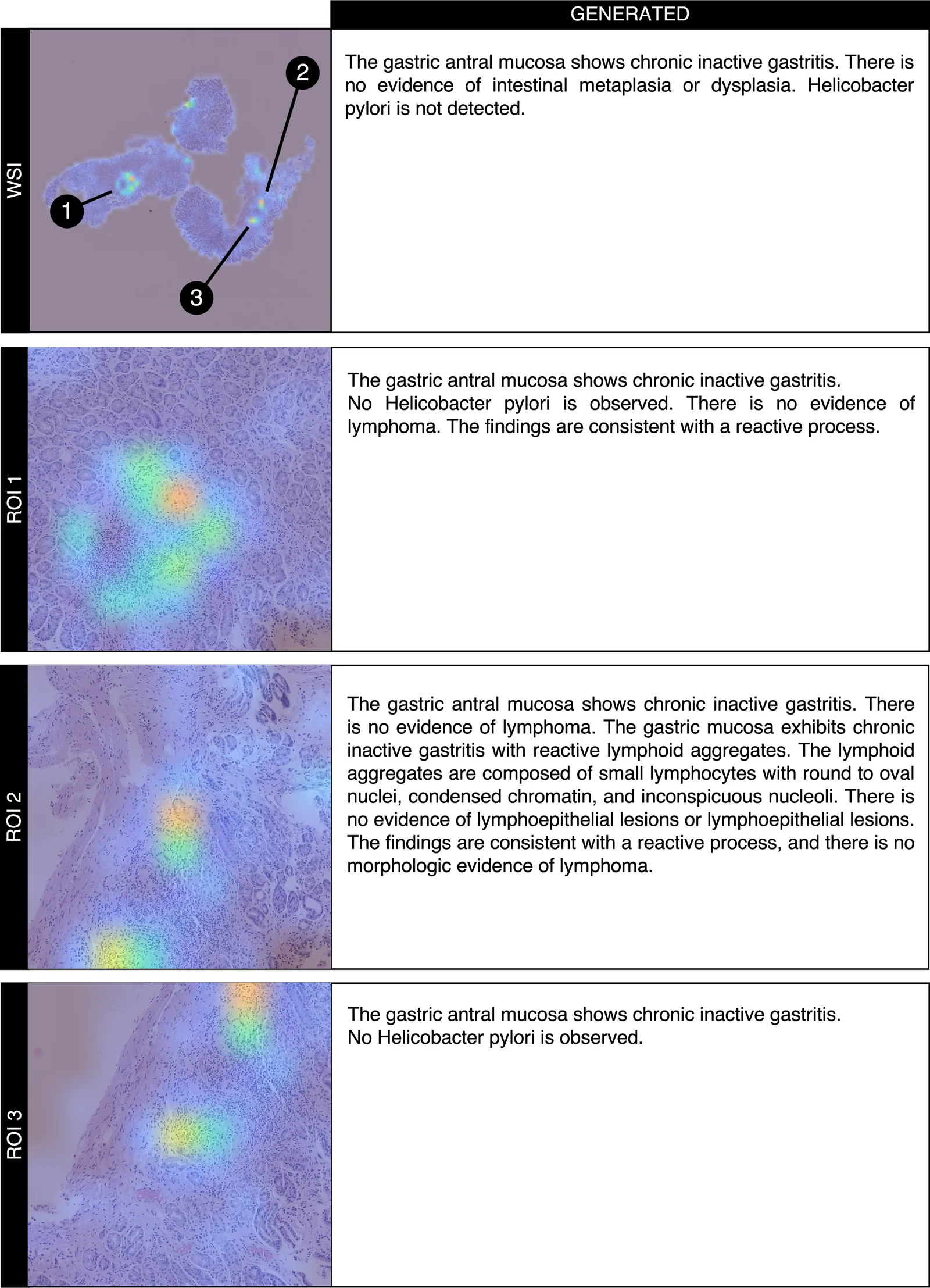
Gastric sample. The slide encoder attention heatmap is shown for a core in a single slide of a gastric specimen, along with the diagnostic report generated by PRISM2. When cropping to each of the three top-attended regions, the PRISM2 report remains consistent with the evidence in the regions of interest.

To qualitatively demonstrate the model’s reporting consistency across highly attended regions, we selected two samples for detailed pathologic review. We evaluate the generated text and compare it with attention maps, the sum of all the scores per tile across attention heads and queries multiplied by the L2-norms of the corresponding values. As shown in Extended Data Figs. 7 and 8, PRISM2 produces verbose specimen descriptions. In both examples, these descriptions remain consistent when cropping to the most attended regions. The attention maps, from the slide encoder’s cross-attention layer, are shown for a single WSI core and each of its top regions of interest.

### Interrogating the data, architecture and optimization design of PRISM2

The performance of PRISM2 is attributable to the sum of data-scale, model architecture and training approach. We designed two studies to assess the effects of these factors. First, we qualitatively review the data processing and PRISM2’s performance on those cases. Second, we perform an ablative analysis, starting from the PRISM data subset and successively building components of PRISM2 to elucidate their impacts. Taken together, these analyses build confidence in the use of language and the importance of scale while highlighting areas for improvement.

PRISM2 training is supervised indirectly by pathology reports. To assess the errors inherent in text preprocessing when preparing training data, and the errors produced by the model after training, we sampled 50 held-out specimens across 10 tissue types (Supplementary Table 2) for review by a pathologist. Error types and counts are detailed in Extended Data Table 1, with examples in Supplementary Note 2.

The ground truth (training data) error rate ranges from 3% for open-ended and multiple-choice questions to 8% for diagnostic summaries and 18% for complementary yes/no questions. This higher rate is driven largely by irrelevant or inaccurate question−answer pairs. Complementary questions are sampled from unrelated specimens, assuming that findings are truly absent from the specimen if not mentioned in its pathology report. Inaccurate questions arise when this assumption fails. Irrelevant questions stem from anatomic sites, margins or disease entities not shared between the specimens.

Despite the breadth of findings in real pathology reports, the PRISM2 model error rate remains low for question answering (7−11%), especially for yes/no questions. On the other hand, it is much higher for diagnostic summaries that must contain all significant findings and have the least specific prompt (that is, ‘Write a report’). Most errors are hallucinations (false positives) or omissions (false negatives), with misclassifications (for example, incorrect staging or tumor grade) dominating for open-ended and multiple-choice questions. Logical inconsistencies, where the model misrepresents a diagnostic finding despite answering correctly elsewhere, are most common in diagnostic summaries.

After investigating the dialogue data, we performed an ablative study, successively adding elements of PRISM2 as described in the ‘Ablation study’ section. Results are presented in Extended Data Table 2 and Supplementary Tables 7−11. Starting with PRISM, we changed the tile embedding model to Virchow2, updated the architecture and prepared more verbose diagnostic report summaries, using only the PRISM training data subset (‘A’ in Extended Data Table 2). This improved diagnostic (0.961 to 0.964 mean AUC) and survival (0.687 to 0.707 C-index) performance. Adding question-answering training data improved prompt-based inference from approximately random (0.498) to 0.653 balanced accuracy (‘B’). Second-stage training with unfrozen Phi-3 Mini weights (‘C’) performed slightly better than PRISM across all measures. We observe that training longer can improve diagnostic performance at the expense of biomarker or survival performance—tasks lacking supervision during training; by freezing the slide encoder (perceiver) weights early (‘D’), we maintain high performance on these tasks with ‘base’ embeddings (0.804 mean biomarker AUC in ‘C’ to 0.811 in ‘D’; 0.697 survival C-index in ‘C’ to 0.700 in ‘D’) while continuing to fine-tune the ‘diagnostic’ embeddings in the language model (achieving 0.965 mean diagnostic AUC, similar to ‘C’). The final PRISM2 uses the full dataset, a 3.5× increase, improving performance across all task categories—most notably diagnostic tasks, where approximately 50% of the improvement over PRISM stems from scale.

## Discussion

A complete pathology foundation model encodes the full range of relevant pathologic semantics not only within tiles but also across WSIs. This minimizes the training and data required to adapt it to downstream tasks while enabling generalization to diverse tasks, such as rare cancer detection and prognostic prediction. In this study, we achieved these aims. PRISM2 base embeddings outperformed those of other slide-level models on diagnostic, prognostic and biomarker tasks. PRISM2 diagnostic embeddings, leveraging a language model trained on diagnostic dialogue, further improved performance on diagnostic tasks. PRISM2 is, to our knowledge, the only model to match or exceed all tested clinical-grade products in cancer detection using prompt-based inference via question answering without further training. Finally, PRISM2 survival embeddings, tuned on nearly 100,000 patients, outperformed base embeddings on prognostic tasks, including RFS and DSS.

Building upon a language model was critical for unlocking PRISM2 prompt-based inference performance. Natural language provides a straightforward way to frame clinical tasks, even when positive or negative cases are difficult to define. For example, contrastive classification for pan-cancer detection requires contrasting all possible cancer-positive and cancer-negative phrases (including benign conditions) across all tissues (Supplementary Tables 5 and 6). Conversely, a single yes/no question (for example, ‘Is malignant tumor present in this specimen?’) can be used across all pan-cancer data. This is less important when the number of candidate labels is small, as in subtyping. However, contrastive approaches do not outperform PRISM2 prompt-based inference in these cases, indicating that PRISM2 allows for general question answering without sacrificing performance on more narrowly defined tasks. Furthermore, prompt-based inference is compatible with multiclass problems—that is, non-mutually exclusive findings within a single specimen. Moving to linear classification removes the need for prompt definition, but PRISM2 still outperforms competing models. This capability comes with an observed tradeoff in generalizability: the diagnostic embedding underperforms the base embedding on tasks not observed during training. In these instances, we recommend using the base embedding.

Optimization details were important for generalizability. We froze the slide encoder before the lowest contrastive validation loss, which improves transferability to biomarker and prognostic tasks. Previous work similarly observed that earlier checkpoints may be more generalizable^41^. We thus rely on the vision−language model, not the slide encoder, to specialize in diagnosis.

We prioritize accurate single-turn question answering over robust chat capabilities. This yields embeddings and responses with exceptional adaptability to downstream tasks by (1) uncalibrated prompt-based inference, (2) calibrating inference probabilities on a validation set and (3) further training on base or diagnostic embeddings. These settings allow adaptation with no additional data (1) or limited additional data ((2) and (3)).

There are still open questions regarding slide-level foundation model advancements, alongside limitations of this work. Because the perceiver-based slide encoder relies on cross-attention rather than self-attention, we omitted position encoding. Interestingly, the CAMELYON17 result draws the necessity of position encodings into question. In clinical practice, lymph node staging requires measuring and counting lesions across nodes. Without global spatial reasoning, PRISM models may be inferring N-stage from local morphological features. Although Virchow2 can encode tiles at various magnifications, PRISM2 used only 0.5 microns-per-pixel. Adopting an aggregator architecture with position encoding and mixed magnification may improve performance on tasks that benefit from greater spatial context, such as cancer subtyping, and may enable measurement tasks, such as mitotic event counting. As evidenced by the modest improvement in biomarker performance compared to baseline models, as well as the large improvement in survival prediction performance gained by fine-tuning on overall survival, further improvement in prognostic and biomarker tasks may require their inclusion during pretraining. This work explored prognostic ability but not counterfactual predictions. Although PRISM2 demonstrates exceptional linear probing performance even when transferring probes across data from different centers, both PRISM2 and its Virchow2 tile embedding model are trained only on slides scanned at MSK, suggesting that further study on robustness may be warranted^42^. Model errors may be reduced by correcting noisy training data and addressing dataset biases. Finally, although full diagnostic report completion is possible, it remains challenging. Grading and subtyping are particularly subjective, and a complete study of report completion would evaluate inter-pathologist variability.

Work in foundation models in computational pathology has moved from the tile level to the slide level^9,11–17^. PRISM2 extends the scale of this effort and is the first, to our knowledge, to achieve clinical-grade cancer detection performance. It is the largest model (4.6 billion parameters) trained on the most comprehensive dataset to date, with nearly 700,000 specimens and clinical reports, covering over 2.3 million slides and 14 million question−answer pairs. Beyond scale, we demonstrate through the inclusion of a large vision−language model that the image−report alignment can be improved by leveraging techniques from the LLM domain. Finally, we found that as diagnostic performance in pathology saturates, further improvements in biomarker and prognostic prediction may be the new research frontier.

## Methods

### Training data

Institutional review board approval was not required for the research described in this study. This study was conducted retrospectively from deidentified data licensed to Paige.AI Inc. from MSK. The data used in this study were all collected originally for clinical use by MSK in the practice setting and are, therefore, considered secondary data. Only data previously deidentified by MSK were utilized in the analysis, and unique patient identifiers were completely removed from the analytical dataset. To our knowledge, MSK has not transferred any data for which the applicable patient has not consented to or otherwise agreed to MSK’s Notice of Privacy Practices or a substantially similar notice, waiver or consent.

The training data comprise 685,507 specimens collected from 200,692 patients. Each specimen is associated with a report and one or more WSIs, totaling 685,507 reports and 2,350,518 slides. The WSIs were formalin-fixed and paraffin-embedded, stained with routine H&E and scanned at ×20, 0.5 microns-per-pixel, using Leica scanners. The data represent a diverse collection of tissues and diagnoses (Fig. 2). Specimens are from MSK (internal) as well as external institutions (submitted, spanning multiple continents); all specimens are scanned, but not stained, and reported at MSK. Each modality is preprocessed to prepare for model training.

Whole-slide tissue segmentation is performed with a trained fully convolutional network developed on pathologist-annotated slides for use with Paige products, with postprocessing via Otsu thresholding with thresholds of (0.4, 0.5). WSIs are processed at ×20 magnification with a size of 224 × 224 pixels, discarding any tiles that are less than 65% tissue. The resulting tiles are then processed by Virchow2 (ref. ^1^) to produce ‘tile embeddings’, the 1,280-dimensional class token output of the model. The distribution of tiles per specimen is very long tailed, as some parts have a large number of WSIs. To prevent training batches from comprising only a few samples, while aiming to not remove specimens, during training, up to 100,000 tile embeddings are loaded per specimen. In cases where specimens have more than 100,000 tile embeddings (approximately 12% of samples in the training dataset), tile embeddings corresponding to WSIs in the specimen are loaded in a random order until the point at which loading all of the tile embeddings for the next WSI would exceed the budget of 100,000. This ensures that partial WSIs are not used. During inference, when batch diversity is no longer a concern, all tiles are loaded. Preprocessing for downstream tasks matches the training preprocessing except for gastrointestinal tasks, where the tissue inclusion criterion is dropped to 10% to enable better coverage of tissue boundaries, which may be important for detecting *Helicobacter pylori*. Raw report data are delivered at the case level. Regular expression (regex) matching is performed to match the report text, which is labeled with part numbers, to the proper set of WSIs, also labeled with part numbers, for that specimen. The reports are a mix of synoptic worksheets and free-text and are stripped of boilerplate content such as headers/footers, disclaimers and repetitious formatting characters.

### Creating clinical dialogue for training

To create high-quality text data for training, we apply GPT-4o^43^ with various prompting strategies to the report data (see Fig. 2 for an example workflow). We create five classes of processed text tasks to be used in training:

1. Clinical report generation. Given a task string (that is, ‘Write a report’), the model must generate the clinical report.
2. Yes/no question answering. Given a binary user question, the model must generate an answer.
3. Open-ended question answering. Given an open-ended user question, the model must provide an answer.
4. Multiple-choice question answering. Given a user question and a list of answer options, the model must select the correct option.
5. Image−text matching. Given a report, the model must identify if the report accurately describes the given WSIs and then generate the correct report if they are mismatched.

We choose these templates to broaden the training distribution beyond the obvious report generation task and enable dialogue-based interactions with the trained model. The process to create the data for each of these tasks is described below, and the full system prompts can be found in Supplementary Note 5.

#### Clinical report rewrites

Using the synoptic reports and free-text notes, we prompt GPT-4o to rewrite all input text, including patient history and specimen description when available, into natural language sentences while retaining all diagnostically relevant findings.

#### Yes/no question−answer pairs

Question−answer pairs are generated through a multistep process. First, GPT-4o is prompted to convert clinical reports into lists of individual findings, similar to MAIRA-2 (ref. ^44^), which are then filtered to exclude molecular and IHC results. These filtered findings are then used as input to GPT-4o with a prompt to generate question−answer pairs for each finding that can be answered with simply ‘Yes’ or ‘No’. Although effective, we found that this process is naturally biased toward creating questions that have the answer ‘Yes’. We expect that this is because negative findings are typically not mentioned in the reports. For instance, a report positively identifying DCIS in breast tissue allows GPT-4o to generate a pair such as: ‘Q: Is DCIS present? A: Yes’. However, a normal breast tissue report does not mention DCIS, so no negative question−answer pair of the form ‘Q: Is DCIS present? A: No’ is generated, biasing the dataset (and resulting model) toward affirmative responses. Inspired by Goyal et al.^45^, to mitigate this bias we implemented a simple method to mine complementary images for additional questions. Specifically, we pair random reports with random questions and prompt GPT-4o to answer based on the provided report. This yields complementary image−question−answer triplets, balancing the data distribution.

#### Open-ended question−answer pairs

Open-ended question−answer pairs are also generated from the list of findings described above. We instruct GPT-4o to create the question−answer pairs such that the questions are not answerable with just yes/no but require more specific answers. Open-ended question−answer pairs help mimic more realistic model interactions and improve the diversity of the training data.

#### Multiple-choice question−option−answer triplets

Multiple-choice question−option−answer triplets are generated from the synoptic reports and free-text notes. We prompt GPT-4.1 (ref. ^46^) to first classify a given report into one of 90 possible CAP Cancer Protocol Templates and then to create as many question−option−answer triplets as possible from the report such that they follow the inferred protocol. We only provide the name of the 90 possible protocols and not the full template as we rely on the inherent recall of these protocols by GPT-4.1.

#### Image−text matching

The matching task uses the clinical report rewrites described above and does not require further preprocessing. This task aims to force the data to use the image information to complete the task.

### PRISM2 architecture

The overall architecture of PRISM2 consists of a slide encoder, a language encoder and a language model, as illustrated in Extended Data Fig. 1 and described below.

#### Slide encoder

The slide encoder uses a perceiver^21^ architecture. The PRISM2 perceiver uses one block, defined as a cross-attention layer between latent queries and tile embeddings followed by six self-attention transformer layers for the latent queries. acGQA^47^ is used in the tile embedding cross-attention. The number of latent queries is set to 256, and an attention pooling layer is used on these to produce the embedding used in the contrastive objective. This embedding is referred to as the ‘base’ embedding. The perceiver and attention pooler have 541 million and 79 million parameters, respectively.

#### Language encoder

BioGPT^22^ is used as the language encoder. We initialize a learnable class token that is concatenated with the input text sequence. The final layer hidden state of the class token is used as the text embedding for the contrastive objective after a linear projection and L2 normalization.

#### LLM decoder

For the dialogue-focused language model component, we use Phi-3 Mini^20^, a 3.8-bilion-parameter decoder-only LLM. We adopt a LLaVa-style^48^ approach to enabling Phi-3 Mini to process both images and text. The 256 latents outputted by the slide encoder are further processed by a two-layer multilayer perceptron (MLP) adapter (29 million parameters) to project them into the Phi-3 embedding space and are inserted into the text token embedding sequence as input to the LLM. This allows Phi-3 to integrate the slide and text information via its self-attention layers. In addition to decoding the language tokens, we also perform analysis with the Phi-3 Mini final layer hidden state of the <∣assistant∣> token. We refer to this as the ‘diagnostic’ embedding.

### PRISM2 training

#### Objectives

PRISM2 is trained with both contrastive and autoregressive objectives. Following refs. ^15,23^, the contrastive loss is

1 $${{\mathcal{L}}}_{{\rm{con}}}=-\frac{1}{N}\left(\mathop{\sum }\limits_{i=1}^{N}\log \frac{\exp ({{v}}_{i}^{\top }{t}_{i}/\tau )}{\mathop{\sum }\nolimits_{j=1}^{N}\exp ({v}_{i}^{\top }{t}_{j}/\tau )}\right.+\left.\mathop{\sum }\limits_{i=1}^{N}\log \frac{\exp ({t}_{i}^{\top }{v}_{i}/\tau )}{\mathop{\sum }\nolimits_{j=1}^{N}\exp ({t}_{i}^{\top }{v}_{j}/\tau )}\right),$$

where *N* is batch size, *τ* > 0 is a learned temperature and (*v*_*i*_, *t*_*i*_) are the image and text representations, respectively, as defined above. Only the diagnostic report rewrites are used as the text input for the contrastive objective. For the autoregressive objective, we apply the standard cross-entropy loss on next-token predictions from Phi-3 Mini. We use the chat templates as the text input and apply the loss only to the assistant regions of the templates:

2 $${{\mathcal{L}}}_{{\rm{chat}}}=-\sum _{t\in {\mathcal{A}}}\log p(\,{y}_{t}|\,{y}_{ < t},{X}),$$

where ${\mathcal{A}}$ is the set of token indices corresponding to assistant tokens and *X* are the image latents after the LLM adapter MLP. The losses are linearly combined to create the total loss:

3 $${{\mathcal{L}}}_{{\rm{total}}}={\lambda }_{{\rm{con}}}{{\mathcal{L}}}_{{\rm{con}}}+{\lambda }_{{\rm{chat}}}{{\mathcal{L}}}_{{\rm{chat}}}.$$

#### Data sampling

For the contrastive objective, report rewrites are randomly sampled from five rewrites per report. For the autoregressive objective, we adopt the Phi-3 (ref. ^20^) chat template using alternating user and assistant tags. The first user message always contains the WSI latents from the slide encoder followed by an initial prompt. In each template, the patient history and specimen description are randomly included, each with a probability of 20%, to allow for conditioning the model on this extra context without being overly reliant on it. Complementary question−answer pairs are included with a sampling rate of 20%. In total, we create 685,000 report rewrites for as many specimens and 14 million question−answer pairs.

#### Optimization

We adopt a two-stage training approach. In the first stage, the slide encoder, attention pooler, LLM adapter and BioGPT weights are updated using the full objective. The Phi-3 Mini weights are constant (frozen) during this stage. We set *λ*_con_ to 0.25 and *λ*_chat_ to 1.0. In the second stage, the contrastive objective is discarded, and the slide encoder weights are frozen. The LLM adapter and Phi-3 Mini are updated to allow the language model to better integrate the perceiver latents and model the language of pathology. The optimizer and learning rate schedules are reset for this stage.

We train the model on 56 A100 40GB GPUs with bf16 precision. We use the AdamW^49^ optimizer with *β*_1_ = 0.9 and *β*_2_ = 0.95 and a learning rate warmup for 500 steps, which is then held constant. We do one epoch of stage 1 training with a post-warmup learning rate of 1.0 × 10^−4^ on all trainable components except the language encoder, which uses a learning rate of 2.0 × 10^−5^ and a 0.1 decay on the slide encoder weights. The large language encoder (Phi-3 Mini) weights are kept frozen in stage 1, but all dialogue objectives are applied. We do 16 epochs of stage 2 training with 500 steps of learning rate warmup to 2.0 × 10^−5^ and no weight decay. The slide and language encoders (perceiver and BioGPT) are frozen, and the large language encoder (Phi-3 Mini) is unfrozen for fine-tuning. An epoch is defined as one pass over each of the four chat templates per specimen, for all specimens in the training data. Because the contrastive objective aligns the image representation with the report and not with each chat template, each specimen is paired with its report four times in a single epoch. Several engineering changes were required for efficient training (see Supplementary Note 6 for details).

#### Survival fine-tuning

The PRISM2 slide encoder was fine-tuned on a large dataset of 225,597 cases with overall survival event data, containing 69,114 death events. Survival prediction is trained by learning a new cross-attention pooling in the (perceiver) slide encoder for ‘survival’ embeddings instead of ‘base’ embeddings and adding a linear regression head on top. This head predicts the log-relative hazard, along with the event time in months, for the partial log-likelihood loss, completing the Cox proportional hazards model. We used the AdamW^49^ optimizer with *β*_1_ = 0.9 and *β*_2_ = 0.98 and a learning rate warmup for 4,000 steps, which is then held constant. We used a post-warmup learning rate of 1.0 × 10^−4^ for the slide encoder and 1.0 × 10^−3^ for the prediction head, with a 0.0001 weight decay. The comparison survival specialist model was trained from scratch using the same architecture with the same hyperparameters as stage 1 PRISM2 training.

### Comparison models

Several slide-level or part-level embedding models were previously reported and are used for comparison. We describe each in brief here. PRISM^15^ is the previous model developed by the PRISM2 team. It differs from PRISM2 in that it is trained using few samples, with fewer types of text outputs and a different training algorithm. TITAN^16^ is a slide-level embedding model that uses vision−language pretraining. TITAN aggregates CONCH^50^ in a three-stage processing using both synthetic captions and medical reports and a training dataset of approximately 340,000 WSIs. COBRA^36^ uses tile representations from four different foundation models to serve as feature space augmentations for a contrastive self-supervised learning approach. COBRA aggregates tiles using a state space architecture and is trained using approximately 3,000 slides. Prov-GigaPath^37^ uses a vision-only approach to creating a slide-level model. Tile embeddings are aggregated using a masked autoencoding approach and a training dataset of approximately 170,000 WSIs. Virchow2 mMean refers to a baseline where the Virchow2 (ref. ^1^) tile embeddings are naively aggregated by simply averaging them all together. Both PRISM2 and COBRA aggregate Virchow2 tile embeddings.

### Evaluation methods

#### Prompt-based inference with question answering

Prompt-based inference with question answering refers to a setting without additional training and includes multiple-choice and yes/no settings. With both yes/no and multiple-choice question answering, we can quantify the probability of predictions. For each classification task, we choose a question prompt *w*_<*i*_ (for example, ‘Is malignant tumor present?’) and consider a set *C* of possible completions *c*. For yes/no question answering, these are ‘Yes’ or ‘No’; for multiple-choice question answering, these are the letter options (for example, ‘A’ and ‘B’). The prompts and corresponding tasks are reported in Supplementary Tables 3 and 4.

Formally, we define our dialogue-based prompt-based inference with generative likelihood scoring as

4 $$f({w}_{ < i},x)=\arg {\mathop{\max }\limits_{c\in C}}\log P({w}_{i}=c| {w}_{ < i},x)-\log P({w}_{i}=c| {w}_{ < i}),$$

where *c* is a completion in a set of completions *C* that cover all classes in the task; *w*_<*i*_ is a tokenized prompt containing a question; and *x* is the context that contains WSI embedding tokens from the slide encoder.

#### Contrastive classification

We evaluate prompt-based inference with PRISM and TITAN using the methodologies reported by the authors, respectively. Generally, both models take the approach of considering the cosine similarity score between the slide embedding **v** and a set of class-specific prompts ${\{{{\bf{t}}}_{c}\}}_{c = 1}^{C}$ and selecting the class that corresponds to the highest similarity:

5 $$f(v)=\arg {\mathop{\max }\limits_{c\in C}}{{\bf{v}}}^{\top }{{\bf{t}}}_{c}$$

The softmax function is used to transform cosine similarities to probabilities, enabling the calculation of AUC. For additional details on the contrastive methodology, see the original publications. The prompts for each task, which are specific to the model, are reported in Supplementary Tables 5 and 6.

#### Linear probe

Beyond prompt-based inference, model embeddings are also evaluated by training a linear classifier or linear regression. To do so, model embeddings are generated, without any text prompting, for training, validation and testing sets of data. Linear classifiers are trained to convergence for each of 24 L2 regularization weights, and the best classifier is selected using the validation set. For survival analysis, a Cox proportional hazards model is fitted to the training set for each of 24 elastic regularization weights (L1 and L2, with an L1:L2 ratio of 0.0001), with the best regressor selected on the validation set.

#### Report completion

We demonstrate the completion of pathology reports with the CAP worksheet for biopsies with invasive carcinoma of the breast^40^. The specimen procedure and laterality fields and the tumor position field are omitted from this analysis, as they constitute sample collection information rather than diagnostic findings derived from H&E-stained slides. The mitotic rate measurement is also excluded as the model was not trained to count. Filling the fields in the CAP template is posed as prompt-based inference, as described earlier in this section, with multiple-choice question answering for multiclass fields (for example, histologic type and histologic grade) and with yes/no question answering for binary and multilabel fields (for example, presence of DCIS or its architectural pattern). The prompts and corresponding tasks are reported in Supplementary Tables 3 and 4. In total, the report can be represented with nine sets of fields, grouped under histologic type, histologic grade, DCIS and ‘other’ (lymphovascular invasion and microcalcifications), filled by asking five multiple-choice questions and nine yes/no questions. We measure the AMR. For multiclass, this is equivalent to adjusted balanced accuracy (adjusted such that random chance is scored as 0).

For prompt-based inference with PRISM2, we infer a prediction probability as the probability of the selected option or of ‘yes’ versus ‘no’. This allows us to evaluate whether the model predictions can be used directly or if they require further calibration, as is often done with clinical-grade products. We evaluate both the AMR of prompt-based inference (observed) and the best possible AMR (‘max AMR’) by calibrating the probability. Calibration is per-class thresholding for binary (and multilabel) tasks or per-class reweighting for multiclass tasks. We find the best thresholds by sweeping thresholds per class and the best reweighting with numerical optimization.

### Evaluation datasets

We evaluate the embeddings of PRISM2 and baseline models on a range of cancer detection, cancer subtyping and molecular tasks described below. For all datasets using MSK data, all patients are held out from the training sets of PRISM2 and Virchow2.

Pan-cancer Detection is an in-house evaluation dataset containing 22,932 H&E WSIs collected from 6,142 specimens. Samples are scanned at MSK and sourced in near-equal quantities from MSK (49%) and WSIs submitted to MSK for review from globally diverse external sites (51%). Of the 16 cancers included in the dataset, seven are rare. The National Cancer Institute (NCI) defines rare cancers as those with an annual incidence in the United States below 15 people per 100,000. Complementing the evaluation dataset, the training and validation sets together contain 89,417 slides across 40,402 specimens and follow a more natural distribution for the frequency of different cancers. Note that the linear classifier trained on the pan-cancer training set is used to evaluate invasive cancer detection via linear probing on the prostate, breast and BLN product benchmarks below.

Product benchmarks are in-house evaluation datasets for the Paige Prostate Detect, Paige Breast and Paige BLN products, which are tissue-specific, clinical-grade models. Each evaluation dataset was curated by pathologists to capture a variety of indications, tumor sizes and surgical methods. More specific diagnoses are grouped into invasive cancer or noninvasive cancer for evaluation. The prostate, breast and BLN datasets contain 2,947 (68% invasive cancer), 1,691 (11% invasive cancer) anf 753 (61% invasive cancer) samples, respectively.

Breast Subtyping is an in-house dataset comprising 11,774 breast H&E WSIs spanning 2,407 specimens. Each specimen is associated with six non-mutually exclusive labels indicating the presence or absence of ILC, IDC, DCIS, lobular carcinoma in situ (LCIS), atypical ductal hyperpalasia (ADH) and atypical lobular hyperplasia (ALH). The dataset is divided at a specimen level into eight folds with a 75/12.5/12.5 percentage split among train, tune and test subsets.

MSK GI is an in-house dataset containing 226,024 gastrointestinal H&E WSIs gathered from 122,601 specimens. The labels span 12 non-mutually exclusive binary classification tasks. The dataset is split into train, tune and test subsets of size 91,276, 17,515 and 13,810 specimens, respectively.

TCGA-BRCA is a publicly available invasive breast cancer (BRCA) dataset focused on discriminating between IDC and ILC. It comprises 1,002 H&E WSIs from TCGA. The data are split into five folds with a 60/20/20 ratio among the train, tune and test subsets for each fold.

TCGA-NSCLC is a publicly available non-small cell lung cancer (NSCLC) dataset composed of 1,043 H&E WSIs from TCGA. Each WSI contains lung adenocarcinoma (LUAD) or lung squamous cell carcinoma (LUSC). The data are split into five folds with a 60/20/20 ratio among the train, tune and test subsets for each fold.

TCGA-RCC is a publicly available renal cell carcinoma (RCC) dataset containing 938 H&E WSIs. Each slide belongs to one of three ground truth subtypes: clear cell renal cell carcinoma (CCRCC), papillary renal cell carcinoma (PRCC) or chromophobe renal cell carcinoma (CHRCC). The data are split into five folds with a 60/20/20 ratio among the train, tune and test subsets for each fold.

CAMELYON16 (refs. ^29,30^) is a public challenge dataset containing 400 H&E lymph node resection WSIs labeled with the presence or absence of cancer. A test set with 130 WSIs is provided, so we perform five-fold the remaining 270 samples to select the linear probe L2 regularization parameter and then retrain to convergence with the selected parameter on the full 270-sample training set.

CAMELYON17 (ref. ^31^) is a public challenge dataset containing 500 H&E lymph node resection WSIs split evenly across 100 patients. A tumor stage (pN0, pN0(i+), pN1mi, pN1 or pN2) is assigned to each patient. The data are sourced from five centers and are split into five folds, one per center, with a 60/20/20 ratio among the train, tune and test subsets for each fold.

PANDA is a public challenge dataset containing 10,616 H&E WSIs with core needle biopsies of prostate cancer. We use the version that excludes slides with noisy labels, resulting in 9,555 WSIs with tumor International Society of Urological Pathology (ISUP) grade of 0 (benign) through 6 (ref. ^32^). These data are sourced from the Radboud University Medical Center and the Karolinska Institute with the source annotated for each sample. We evaluate both a mixed-institution version of this dataset, ‘PANDA-MI’, and a separated-institution version, ‘PANDA-SI’, that requires transferring the linear probe learned on one source to the other. For PANDA-MI, we use a 70/15/15 ratio among the train, tune and test subsets with 6,686, 1,430 and 1,439 WSIs, respectively. For PANDA-SI, we do two-fold cross-validation with one data source per fold.

IMP-CRS^33–35^ is a public dataset containing 5,333 colorectal biopsy and polypectomy H&E WSIs, retrieved from the data archive of IMP Diagnostics laboratory. All slides are labeled with tumor grades: non-neoplastic, low-grade lesion and high-grade lesion. Because a test set of 900 WSIs is provided, we split the remaining 4,433 samples into a 3,533 training slides and 900 tuning slides.

MSK biomarker prediction is an in-house dataset containing 37,633 WSIs gathered from 33,949 samples. The ground truth labels span 10 different biomarkers: Prostate AR, Ovarian FGA, Esophagogastric Her2, Colorectal MSI, Lung EGFR, Melanoma BRAF, Bladder FGFR, Breast CDH1, Endometrial PTEN and Breast PIK3CA. These labels were originally identified by MSK-Integrated Mutation Profiling of Actionable Targets (MSK-IMPACT) sequencing^51^, a targeted test for genetic mutations. The dataset is split into train, tune and test subsets of size 22,634, 5,606 and 5,709 specimens, respectively. Each label corresponds to an oncogenic mutation associated with a cancer, with both primary and metastatic samples present in the dataset. AR in prostate cancer is positive when the copy number amplification of androgen receptor (AR) is two-fold or higher. FGA in high-grade ovarian cancer, characterized by presence of TP53 and genome instability with widespread genetic alteration, is fraction of genome altered (FGA) positive where FGA ≥30%. Her2 overexpression and/or amplification in esophagogastric cancer, associated with possible anti-human epidermal growth factor receptor 2 (HER2) antibody therapy, is determined with HER2 IHC 2+/FISH+ or IHC 3+. Microsatellite instability (MSI) in CRC occurs when DNA regions with short, repeated sequences (microsatellites) are disrupted by single-nucleotide mutations due to the inactivation of any mismatch repair (MMR) gene, which would otherwise correct these mutations. MSI high and/or deficient mismatch repair (dMMR) are identified with IHC and MSK-IMPACT sequencing, prioritizing IHC results when both test outcomes are available. Epidermal growth factor receptor (EGFR) in lung cancer, fibroblast growth factor receptor (FGFR) alteration in bladder cancer, PIK3CA oncogenic mutation in breast cancer and oncogenic mutation of the phosphatase and tensin homolog (PTEN) tumor suppressor gene in endometrial cancer are considered positive for the presence of point mutations in the genes with any oncogenic effect (including predicted/likely/oncogenic) based on OncoKB annotation^52^. BRAF mutation status in melanoma is determined by the presence of a V600E mutation. The biallelic alterations affecting cadherin-1 (CDH1) in breast cancer are determined by inactivating mutations associated with loss of heterozygosity or a second somatic loss-of-function mutation as determined by MSK-IMPACT.

TCGA biomarker prediction is a public dataset containing 10,493 WSIs gathered from 8,611 samples. The ground truth labels are a subset of those in the MSK biomarker prediction dataset: Colorectal MSI, Lung EGFR, Melanoma BRAF, Bladder FGFR, Breast CDH1, Endometrial PTEN and Breast PIK3CA. Training for each label is done with three-fold cross-validation. Each label corresponds to an oncogenic mutation associated with a cancer, with only primary samples present in the dataset.

MSK CRC RFS is an in-house dataset of CRC cases curated for recurrence analysis and RFS prediction. It contains 884 recurrence events across 1,260 cases, spanning 6,513 WSIs. Evaluation on these data is done with five-fold cross-validation, using month-level precision for the time to event.

TCGA CRC DSS is a set of 13 tasks derived from the public TCGA dataset spanning 9,041 cases with 11,072 WSIs and 2,577 disease-specific death events. DSS is evaluated with five-fold cross-validation for each of the following tasks: CRC (colorectal cancer: COAD and READ), NSCLC (non-small cell lung cancer: LUAD and LUSC), RCC (renal cell carcinoma: KIRK, KIRP and KICH), BRCA (breast cancer), CNS (central nervous system: GBM and LGG), SARC (sarcoma), ENDO (endometrial: UCEC and UCS), LBTC (liver and biliary tract cancer: LIHC and CHOL), MEL (melanoma: SKCM and UVM), HNSC (head and neck cancer), BLCA (bladder cancer), GI (upper gastrointestinal tract: ESCA and STAD) and PAAD (pancreatic cancer). Tasks were curated such that they have at least 100 DSS events each.

Invasive Breast Cancer Pathology Report Completion is an in-house dataset of 1,638 invasive breast cancer biopsy specimens and their associated pathology reports. The pathology reports are standardized to match the current CAP ‘Breast Biopsy, Invasive’ cancer protocol template. Specimens were selected to ensure that the histologic type of the invasive cancer is represented by one of the closed-ended options in the protocol. Macroscopic and quantitative protocol fields were omitted from evaluation. Specifically, procedure type, specimen laterality, tumor site, mitotic rate and tumor measurement were not included.

Metrics. For linear probing, we use (one-versus-one) AUC as the primary metric so that model performance could be evaluated without tuning a threshold for the predicted class probabilities. On the other hand, we primarily evaluate prompt-based inference with balanced accuracy by assuming a threshold at 0.5 probability. For question answering, this is equivalent to taking the model responses directly, without calibration. For fairness, we use product-specific thresholds for clinical-grade products, as these were carefully tuned during product development. Balanced accuracy is the mean recall across all classes, which makes it a suitable metric for imbalanced data.

Statistical analysis. Statistical significance was assessed using two-sided paired permutation tests with 1,000 permutations. Confidence intervals of 95% were estimated via bootstrapping using 1,000 samples. In cases where prompt-based inference is also reported, metric computation (and, thus, statistical analyses) is done with the micro-average across folds to enable direct comparison (that is, no class reweighting). For TCGA DSS, TCGA biomarkers and MSK biomarkers, the macro-average is used.

### Ablation study

To understand the effects of various optimization and data decisions, we designed an ablation study. Using PRISM as a reference starting point, we sequentially added aspects of PRISM2, building up to the final model by scaling the data. For each setting, we report the performance on four categories of tasks: diagnostic (pan-cancer detection and cancer, precursor and benign condition detection in the breast with MSK data as well as BRCA and NSCLC subtyping with TCGA data), biomarker (MSK and TCGA datasets), survival (TCGA datasets) and prompt-based inference (MSK pan-cancer, MSK breast, TCGA BRCA and TCGA NSCLC datasets). We letter each setting (A)–(D), as also described in Extended Data Table 2. Specifically, starting with the PRISM data subset of 195,000 reports, we train a model that uses the PRISM2 format of report rewrites, Virchow2 tile embeddings (PRISM used Virchow) and the PRISM2 architecture (ablation A). Differing from PRISM, the PRISM2 architecture swaps the BioGPT encoder−decoder model with the Phi-3 Mini LLM, drops cross-attention between the perceiver and the language model in favor of feeding the perceiver ‘latent’ tokens as input to the language model and modifies the perceiver configuration. We then train the following additional settings, which accumulate: (ablation B) add the additional chat templates—that is, question answering and image−report matching; (ablation C) continue training with a second stage where the language model is unfrozen and fine-tuned (after four epochs, roughly equivalent to one epoch on the full PRISM2 dataset); and (ablation D) freeze the perceiver weights when starting stage 2. The final configuration is the presented PRISM2 model, where we scale the data (approximately 3.5×) from the PRISM subset to the full dataset used to train PRISM2. Model checkpoints were selected for evaluation by lowest epoch-wise validation loss. We used linear probing to evaluate diagnostic, biomarker and survival performance. We used diagnostic embeddings for diagnostic tasks, when available (C, D), and base embeddings otherwise (A, B), as well as for biomarker and survival tasks. We used question answering for prompt-based inference, except with PRISM and TITAN, where we used contrastive prediction.

### Software

All data processing, model training and analysis were performed using Python (version 3.10) and open-source libraries. For data curation, we used Pandas (version 2.2.2) for metadata management and OpenSlide (version 1.3.1) and Pillow (version 10.0.0) for WSI preprocessing and image decoding. Specimen-level and report-level metadata were managed through internal Paige libraries. All model development and experiments were conducted using PyTorch (version 2.3.1) and PyTorch Lightning (version 2.3.0) as the core deep learning frameworks. We used the publicly available Virchow2 model (https://huggingface.co/paige-ai/Virchow2) for WSI embedding generation. We used torchsurv (version 0.1.4) for survival-specific objectives. Additional scientific libraries included scikit-learn (version 1.4.2), einops and nltk (version 3.9). Dialogue generation and evaluation were implemented through the Paige internal reporting framework using the OpenAI API (OpenAI version 0.28.1) with the GPT-4o model.

### Reporting summary

Further information on research design is available in the Nature Portfolio Reporting Summary linked to this article.

## Online content

Any methods, additional references, Nature Portfolio reporting summaries, source data, extended data, supplementary information, acknowledgements, peer review information; details of author contributions and competing interests; and statements of data and code availability are available at https://doi.org/10.1038/s41591-026-04521-4.

## Supplementary information

Supplementary Information Notes 1−10, containing Tables 1−24 and acronyms.

Reporting Summary

## Figures and Tables

**Extended Data Table 1:**
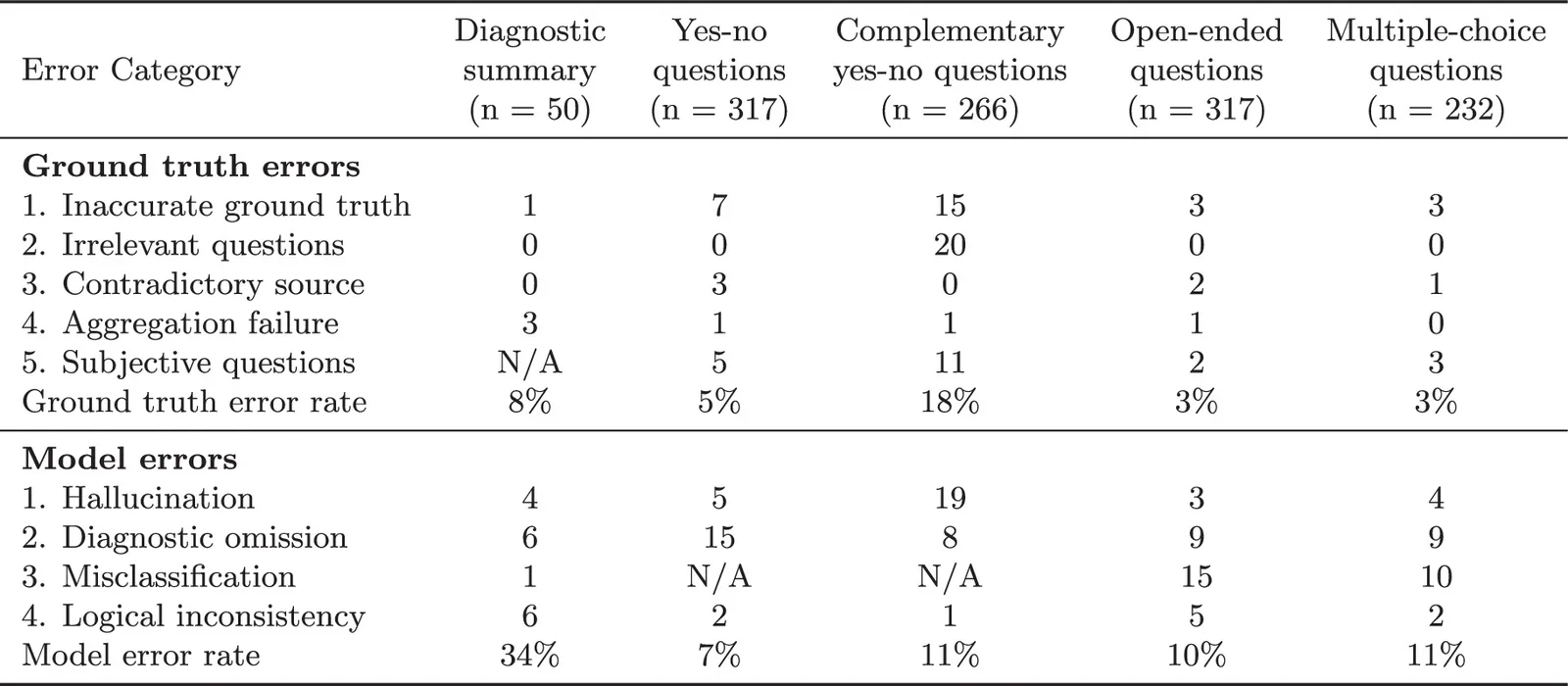
Qualifying and quantifying the errors

Qualifying and quantifying the errors in ground truth text templates generated with GPT-4o (‘ground truth errors’) and PRISM2 responses to the corresponding prompts (‘model errors’), as evaluated by a pathologist.

**Extended Data Table 2:**
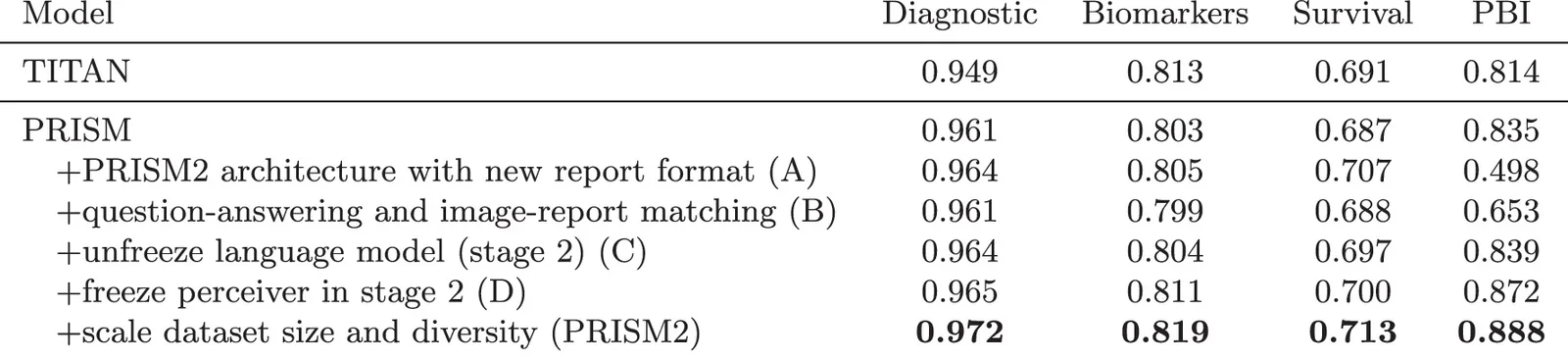
Ablation study

Ablation study of the cumulative effects of optimization and data decisions. Starting with the reference point of PRISM, we systematically measure the effects on downstream performance, reporting the average AUC for diagnostic and biomarker tasks, average c-index for survival, and average balanced accuracy for prompt-based inference (PBI) prediction. We include the results for TITAN as well for reference. Highest scores shown in bold.

## Data Availability

This study did not specifically collect patient data. The retrospective analysis utilized proprietary deidentified digital pathology whole slides and associated metadata that were exclusively licensed by Paige.AI Inc. from Memorial Sloan Kettering Cancer Center. Requests for data need to be submitted to Paige.AI (https://paige.ai/contact-us/) and evaluated by Paige.AI and Memorial Sloan Kettering Cancer Center on a case-by-case basis. Paige.AI will aim to provide an initial response within 90 calendar days of receipt of a complete request. All requests complying with the internal policies of Paige.AI and Memorial Sloan Kettering Cancer Center will be granted. All TCGA data used in this study are publicly available through the NCI Genomic Data Commons portal at https://portal.gdc.cancer.gov/. The CAMELYON16 and CAMELYON17 datasets are publicly available via the Grand Challenge platform (https://camelyon16.grand-challenge.org/Data/ and https://camelyon17.grand-challenge.org/Data/). The PANDA challenge dataset is available via Kaggle at https://www.kaggle.com/c/prostate-cancer-grade-assessment/data. The IMP-CRS dataset is publicly accessible via the INESC TEC Research Data Management repository at https://rdm.inesctec.pt/dataset/nis-2023-008. To facilitate reproducibility, all pre-generated PRISM2 and baseline model embeddings and dataset tables are available via Zenodo at https://doi.org/10.5281/zenodo.19056118 (ref. ^53^).

## Code availability

The full PRISM2 model weights are publicly available for research use through Hugging Face at https://huggingface.co/paige-ai/Prism2 and https://huggingface.co/paige-ai/Prism2-survival, along with example code for pathology image-based question answering and the generation of image embeddings. The PRISM2 training and inference pipelines were developed using internal Paige.AI and Microsoft Research infrastructure and cannot be fully released due to dependencies on proprietary libraries, distributed compute environments and commercial intellectual property complexities. To facilitate reproducibility, linear probing evaluation code is available via Zenodo at https://doi.org/10.5281/zenodo.19056118 (ref. ^53^). All experiments are documented in the Methods section to enable independent replication. PRISM2 builds upon open-source components including the PyTorch (https://github.com/pytorch/pytorch) and PyTorch Lightning (https://github.com/Lightning-AI/pytorch-lightning) frameworks.
