# Supplementary material for: End-to-end multimodal pathology foundation model with clinical dialogue
Source: Nat Med. 2026 Jul 31;32(9):3203–13. doi: 10.1038/s41591-026-04521-4 (PMC13577907; doi:10.1038/s41591-026-04521-4)
Supplement: Supplementary file 1 [file 41591_2026_4521_MOESM1_ESM.pdf]

nature medicine

Supplementary information https://doi.org/10.1038/s41591-026-04521-4

End-to-end multimodal pathology foundation model with clinical dialogue

In the format provided by the authors and unedited

S Supplementary Notes

S.1 Summary of slide-level and language-based foundation models in histopathology

Model	Data size		Objective function	Notes
	WSI	Reports		
**PRISM2**	2.35M	685K	Adapted CoCa	This work.
TITAN [16]	336K	183K	Adapted CoCa	TITAN uses embeddings from the pretrained CONCH [50] embeddings and three stages of training. In addition to pathology reports, synthetic question-answer pairs are used.
PRISM [15]	587K	183K	CoCa	Unlike PRISM2, PRISM uses short pathology rewrites and the bioGPT model.
Prov-Gigapath [9]	172K	–	MAE	Prov-Gigapath uses an image-only masked autoencoding objective with dilated attention [53] to allow for long sequence lengths.
COBRA [36]	3.05K	–	Contrastive loss	COBRA slide encoder uses a MAMBA-2-based architecture [54] and different foundation model embeddings and magnifications to create augmentations for use in contrastive training.
CHIEF [17]	60.5K	–	CLIP	CHIEF uses an image aggregator that aligns slide-level representations to text embeddings based on anatomical site.

Supplementary Table S1: A summary of slide-level foundation models. MAE [55]: masked autoencoder, CoCa [23]: contrastive captioner, CLIP [56]: contrastive language-image pre-training

S.2 Qualifying and quantifying errors in reference chat templates and model text predictions

PRISM2 training is supervised indirectly by pathology reports. Instead of using raw reports, we pre-processed the reports to produce diagnostic report summaries and various question-answer pairs, as described in Sec. 4.2. To qualify and quantify the errors inherent in this conversion, as well as the errors produced by the model after training, we sampled 50 specimens across 10 tissue types, for review by a pathologist. The data distribution is shown in Supplementary Tab. S2. The identified error types, along with examples, are specified thereafter.

Tissue	No. cancer	No. precursor	No. benign
Breast	3	1	1
Prostate	3	1	1
Lung	3	1	1
Colon	3	1	1
Skin	3	1	1
Lymph node	4	0	1
Bone marrow	3	1	1
Kidney	3	1	1
Thyroid	4	0	1
Ovary	3	1	1

Supplementary Table S2: The data distribution of the 50 specimens sampled for an error analysis of all generated text templates and their associated model predictions.

S.2.1 Reference text errors

Inaccurate Ground Truth. The reference text contradicts the source pathology report or visual evidence. *Example*: the reference text notes the absence of biopsy site changes, hemosiderin deposition, or reactive pneumocytes when they are visible to the reviewing pathologist. *Example*: reference text notes that malignant prostatic tissue is absent or that multinodular hyperplasia exists in breast resections, contrary to visual evidence.

Irrelevant Questions. The generated questions ask about anatomical sites, margins, or disease entities that have nothing to do with the associated specimen. *Example*: the reference is a metastatic prostatic adenocarcinoma in a lymph node excision, therefore margin-specific questions regarding the eustachian tube, tongue base, and pterygoid fat are irrelevant.

Contradictory Source. The source pathology report contains clerical errors or impossibilities. *Example*: the report for a gynecologic specimen describes a "well-differentiated neuroendocrine tumor" while simultaneously describing a high mitotic rate and high-grade morphology. *Example*: a report states that the bilateral ovarian surfaces are heavily involved by high-grade serous carcinoma but then concludes that the ovaries are free of tumor.

Aggregation Failure. Findings are derived from the gross description or another specimen, rather than from the description of the specimen of interest. *Example*: the report summary or question-answer pair describes gross staging metrics, classifying a clear cell renal cell carcinoma as pT3a and noting that it extends macroscopically beyond Gerota's fascia, which would place it in the pT4 stage. Other reference text records exact macroscopic tumor measurements, like an 8.7cm diameter, or the status of regional lymph nodes that are present in different paraffin blocks not included with the provided specimen.

Subjective Questions. Questions that use subjective terminology or require clinical history to answer. *Example*: some questions ask the model to score the presence of "mild chronic lymphocytic thyroiditis" or "reactive epithelial changes" which suffer from high inter-observer variability even among expert

pathologists. Other questions inquire about vague spatial relationships, such as whether a submucosal neuroendocrine tumor is located "near" an adjacent adenocarcinoma.

S.2.2 Model prediction text errors

Hallucination. The model invents diagnostic features, TNM staging data, or pathological findings that are absent. Also called a false positive. *Example*: in a gynecologic specimen, the model invents findings like adenomyosis or leiomyomas that are absent in the specimen.

Diagnostic Omission. The model fails to identify a pathological feature or invasion type that is visibly present and noted in the reference. Also called a false negative. *Example*: the model overlooks critical findings in thyroid follicular neoplasms such as angioinvasion or extrathyroidal extension into capsule or skeletal muscle, despite identifying the correct histologic subtype. *Example*: in a breast specimen, the model fails to identify focal ductal carcinoma in situ alongside invasive lobular morphology. *Example*: in colorectal a specimen, the model misses subtle metastatic adenocarcinoma deposits within pericolorectal soft tissue.

Misclassification. The model incorrectly assigns the grade, histologic subtype, or architectural pattern of a lesion. *Example*: sebaceous carcinoma in lymph node metastases is mistaken for squamous cell carcinoma. *Example*: in a breast biopsy, solid ductal carcinoma in situ is mischaracterized as having a micropapillary architectural pattern. *Example*: in an appendiceal neuroendocrine tumor, a high grade (G3) is incorrectly assigned to a well-differentiated (G1) appendiceal neuroendocrine tumor.

Logical Inconsistency. The model produces an incorrect response when it has also output the correct information with a different prompt. *Example*: in a thyroid carcinoma, the model identifies the presence of focal tumor necrosis and affected surgical margins in targeted questioning, only to declare the absence of these features in its diagnostic summary report. *Example*: it accurately identifies extensive spread through air spaces (STAS) in NSCLC or positive viable tumor cells in one prompt but misses these findings in another prompt.

S.3 Prompt-based inference: dialogue prompts and completions

Tables S3 and S4 contain the prompts used in prompt-based inference and their completions for yes-no and multiple choice questions, respectively.

Task Class	Task	Prompt
MSK Pan-cancer	Cancer Detection	Is malignant tumor identified in the specimen?
MSK Breast Subtyping	Invasive ductal carcinoma	Is invasive ductal carcinoma (IDC) identified in the specimen?
	Invasive lobular carcinoma	Is invasive lobular carcinoma (ILC) identified in the specimen?
	Ductal carcinoma in situ	Is ductal carcinoma in situ (DCIS) identified in the specimen?
	Lobular carcinoma in situ	Is lobular carcinoma in situ (LCIS) identified in the specimen?
	Atypical ductal hyperplasia	Is atypical ductal hyperplasia (ADH) identified in the specimen?
	Atypical lobular hyperplasia	Is atypical lobular hyperplasia (ALH) identified in the specimen?
	Invasive carcinoma	Is invasive carcinoma identified in the specimen?
	Benign or unremarkable	Is the specimen benign or unremarkable?
MSK GI Suite	Adenocarcinoma	Is adenocarcinoma identified in the specimen?
	Lymphoma	Is lymphoma identified in the specimen?
	Metastatic Tumor	Is metastatic tumor identified in the specimen?
	Neuroendocrine Tumor	Is neuroendocrine tumor identified in the specimen?
	Squamous Cell Carcinoma	Is squamous cell carcinoma identified in the specimen?
	Tumor Differentiation	Is malignant tumor identified in the specimen?
	Dysplasia	Is dysplasia identified in the specimen?
	Polyp	Is polyp identified in the specimen?
	No Significant Abnormalities	Is there no significant abnormalities identified in the specimen?
	H. Pylori Helicobacter	Is h pylori helicobacter identified in the specimen?
	Barretts Esophagus	Is intestinal metaplasia (Barrett's esophagus) identified in the specimen?
	Other Inflammatory Condition	Is inflammatory condition identified in the specimen?
Report filing	Ductal carcinoma in situ	Is ductal carcinoma in situ (DCIS) identified in the specimen?
	DCIS cribriform architecture	Does ductal carcinoma in situ show cribriform architectural pattern?
	DCIS micropapillary architecture	Does ductal carcinoma in situ show micropapillary architectural pattern?
	DCIS papillary architecture	Does ductal carcinoma in situ show papillary architectural pattern?
	DCIS solid architecture	Does ductal carcinoma in situ show solid architectural pattern?
	Vascular invasion	Is lymphovascular or vascular invasion identified in the specimen?
	Microcalcifications	Are microcalcifications present in the specimen?
	Microcalcifications	Are microcalcifications present in ductal carcinoma in situ?
	Microcalcifications	Are microcalcifications present in invasive carcinoma?
	Microcalcifications	Are microcalcifications present in non-neoplastic tissue?

Supplementary Table S3: Prompts for tasks posed as yes-no questions. Prompt-based inference is performed by scoring the completion options: {Yes, No}. See eqn. 4 for the scoring function.

Task Class	Task	Prompt
TCGA Subtyping	TCGA-BRCA	What is the histologic type?<br>A. Invasive ductal carcinoma<br>B. Invasive lobular carcinoma
	TCGA-NSCLC	What is the histologic type?<br>A. Adenocarcinoma<br>B. Squamous cell carcinoma
	TCGA-RCC	What is the histologic type?<br>A. Clear cell renal cell carcinoma<br>B. Papillary renal cell carcinoma<br>C. Chromoprobe renal cell carcinoma
Report filing	Histologic type	What is the histologic type?<br>A. No residual invasive carcinoma<br>B. Invasive ductal carcinoma of no special type<br>C. Micro-invasive carcinoma<br>D. Invasive lobular carcinoma<br>E. Invasive carcinoma with mixed ductal and lobular features<br>F. Invasive carcinoma with features of (specify)<br>G. Tubular carcinoma<br>H. Invasive cribriform carcinoma<br>I. Mucinous carcinoma<br>J. Invasive micropapillary carcinoma<br>K. Apocrine adenocarcinoma<br>L. Metaplastic carcinoma<br>M. Encapsulated papillary carcinoma with invasion<br>N. Solid papillary carcinoma with invasion<br>O. Intraductal papillary adenocarcinoma<br>P. Adenoid cystic carcinoma<br>Q. Neuroendocrine tumor<br>R. Neuroendcrine tumor<br>S. Invasive carcinoma, type cannot be determined<br>T. Other histologic type not listed
	Tubule formation	What is the tubule formation score?<br>A. Score 1<br>B. Score 2<br>C. Score 3
	Nuclear pleomorphism	What is the nuclear pleomorphism score?<br>A. Score 1<br>B. Score 2<br>C. Score 3
	Nuclear grade	What is the nuclear grade of DCIS?<br>A. Low<br>B. Intermediate<br>C. High

Supplementary Table S4: Prompts for tasks posed as multiple choice questions. Prompt-based inference is performed by scoring the completion options: {A., B., etc.} where the set depends on the question formulation. See eqn. 4 for the scoring function.

S.4 Contrastive prediction prompts

Tables S5 and S6 contain the contrastive prediction prompts for TITAN and PRISM, respectively.

Task	Class	Prompts
TCGA-NSCLC	LUAD	adenocarcinoma<br>lung adenocarcinoma<br>adenocarcinoma of the lung<br>LUAD
	LUSC	squamous cell carcinoma<br>lung squamous cell carcinoma<br>squamous cell carcinoma of the lung<br>LUSC
TCGA-BRCA	IDC	invasive ductal carcinoma<br>breast invasive ductal carcinoma<br>invasive ductal carcinoma of the breast<br>invasive carcinoma of the breast, ductal pattern breast IDC
	ILC	invasive lobular carcinoma<br>breast invasive lobular carcinoma<br>invasive lobular carcinoma of the breast<br>invasive carcinoma of the breast, lobular pattern breast ILC
TCGA-RCC	CCRCC	clear cell carcinoma<br>renal clear cell carcinoma<br>clear cell renal cell carcinoma<br>clear cell RCC<br>CCRCC
	PRCC	papillary renal cell carcinoma<br>renal cell carcinoma, papillary type<br>papillary RCC<br>PRCC
	CHRCC	chromophobe renal cell carcinoma<br>chromophobe RCC<br>chromophobe carcinoma<br>renal cell carcinoma, chromophobe type<br>CHRCC
MSK Pancancer	Positive	cancer<br>carcinoma<br>adenocarcinoma<br>malignant<br>metastatic<br>invasive<br>sarcoma
	Negative	benign<br>inflammation<br>normal<br>infectious<br>cyst<br>polyp<br>unremarkable

Supplementary Table S5: Class prompts for tasks posed as contrastive alignment for TITAN. For TCGA tasks, we use the prompts published by TITAN [16]. For cancer detection, TITAN did not publish prompts nor address this task so we made reasonable selections. During evaluation we follow the authors' prompting and ensembling methodology. The publicly released TITAN model code embeds each prompt in a series of templates as described in [16].

Task	Class	Prompts
TCGA-NSCLC	LUAD	adenocarcinoma of the lung (LUAD)
	LUSC	squamous cell carcinoma of the lung (LUSC)
TCGA-BRCA	IDC	invasive ductal carcinoma (IDC)
	ILC	invasive lobular carcinoma (ILC)
TCGA-RCC	CCRCC	clear cell renal cell carcinoma (CCRCC)
	PRCC	papillary renal cell carcinoma (PRCC)
	CHRCC	chromophobe renal cell carcinoma (CHRCC)
MSK Pancancer	Positive	cancer<br>carcinoma<br>adenocarcinoma<br>malignant<br>metastatic<br>invasive<br>sarcoma
	Negative	benign<br>inflammation<br>normal<br>infectious<br>cyst<br>polyp<br>unremarkable

Supplementary Table S6: Prompts for tasks posed as contrastive alignment for PRISM [15]. During evaluation we follow the authors' prompting and ensembling methodology. For simplicity, prompts are kept concise and are not embedded in templates.

S.5 GPT-4o rewriting and question-answer pair generation prompts

Clinical history, specimen, and report rewriting prompt:

```
# Role

You are a pathology assistant whose goal is to help rewrite pathology reports in order to be used for in
    training a whole slide image captioning AI model.

# Instruction

You are given a pathology report describing a tissue sample and sometimes a clinical history and description
    of the specimen under review. You need to rewrite the report as if you're examining the sample yourself
    using natural language. Follow these instructions carefully and to the letter:

- Rewrite the entire report in natural language in the order of appearance (i.e. clinical history -> specimen
    description -> diagnosis).
- Separate the sections into their own key/value pairs: "history", "specimen", "diagnosis".
- If any section is not present, fill with null value.

## History section instruction
Use the information from the "History" section provided by the user to fill in the "history" section of your
    output. Follow these instructions carefully to do this:

- Never include dates and times or names of people/doctors/clinics.
- Do not mention if the patient had previous appointments.
- If age and/or gender of the patient is provided, retain that information in your rewrite.

## Specimen section instruction
Use the information from the "Specimen" section provided by the user to fill in the "specimen" section of your
     output. Follow these instructions to achieve that:

- Rewrite the specimen description to capture specimen type and procedure used to obtain the specimen and any
    other relevant information.

## Diagnosis section instruction
Use the information from the "Diagnosis" and "Addenda" sections provided by the user to fill in the "diagnosis
    " section of your output. You must follow these instruction with extreme attention to detail and care:

- Start with the most clinically significant finding followed by the findings of lesser significance.
- Include relevant morphological details that can be observed from a whole slide image, such as cell types,
    structures, and patterns if they are provided.
- Do not mention anything related to the patient's history or prior samples or material.
- Do not any specific macroscopic locations within the specimen (i.e. exclude things like right, left, apex,
    upper quadrant, mid to base, etc.).
- Do not mention the number of specimens examined (i.e. don't talk about number of lymph nodes inspected)
- Only talk about what could be inferred from looking at a whole slide image of the specimen.
- Exclude results or mentions of immunohistochemical (IHC) studies, molecular assays, or in situ hybridization
    .
- Never mention physical measurements or quantities using mm or cm or inches, etc.
- Only mention the results that are obvious from the report.
- Include relevant info from "Note" or "Addenda" that follows these instructions.
- Do not use language that references the given report.
- The language should read as if you're a pathologist inspecting the specimen and actively diagnosing the
    sample.

# Examples

## Example of a diagnosis rewrite that properly excludes macroscopic locations
~~~
User: Diagnosis: Metastatic carcinoma involving right ovary and left fallopian tube, unremarkable left ovary
    and right fallopian tube
Assistant: "diagnosis": "Metastatic carcinoma is observed in the ovaries and fallopian tube."
~~~
```

````
## Examples of properly excluding immunohistochemistry (IHC) information in diagnosis
```
User: Diagnosis: Invasive Carcinoma Ductal, NOS type. Progesterone Receptor Negative (no nuclear staining).
    HER2 Negative (0).
Assistant: "diagnosis": "There is invasive ductal carcinoma present."
```

## Example of a diagnosis rewrite that properly omits quantifying number of specimens examined
```
User: Diagnosis: Lymph Nodes Number with metastasis: 2
Assistant: "diagnosis": "Metastasis is present in lymph nodes."
```

## Example of a diagnosis rewrite that properly omits mention of a patient's prior samples
```
User: Diagnosis: Metastatic mammary carcinoma, morphologically similar to the patient's
prior specimen.
Assistant: "diagnosis": "Metastatic mammary carcinoma is detected."
```

## Example of a diagnosis rewrite that properly excludes measurements
```
User: Diagnosis: Keratinizing dysplasia, moderate.  Depth of invasion is 9 mm.
Assistant: "diagnosis": "Examination reveals moderate keratinizing dysplasia."
```

# Output
Generate five distinct summaries with a variety of linguistic styles with proper punctuation but still
    accurate to the provided information. Output the resulting 5 summaries as a list in JSON format. Don't
    output anything else. Do not wrap the output in markdown. The JSON structure should be formatted like:

{reports: [{"history": ..., "specimen": ..., "diagnosis":...}, ...]}
````

List of findings prompt:

```
You are an AI pathology assistant. You are helping process reports from whole slide images.

Please extract phrases from the pathology report which refer to tissue, diagnostic findings, margins, or prior
     treatment visible in a histological whole slide image, or the absence of such.

Rules:
- Break down the report into multiple findings
- List the findings in multiple lines
- Each unique finding in the report has its own line
- Each line contains only one unique finding
- Findings are written in one sentence in naturally-sounding language
- Sentences are short and contain only one unique histological finding
- If there are more findings in one line, split them into multiple lines with one finding per line
- Findings don't contain numbers
- Only keep findings that are related to visible features on a histological whole slide image
- Exclude physical measurements (e.g. "...1.9 cm") or percentages (e.g. "...5%").
- Exclude macroscopic locations (e.g. "left", "right", "sigmoid").
- Exclude quantities referring to number of slides or specimens examined (e.g. "5 lymph nodes", "3 consecutive
     slides")
- Exclude immunohistochemical (IHC) and molecular tests.

The objective is to extract phrases which refer to things which can be located on a histological whole slide
    image, or confirmed not to be present.
```

Yes-No question-answer pair generation prompt:

````
You are a pathology instructor at a world class medical school. Create one yes and one no question/answer pair
     for each line in a given report. Do not reference the given report in your wording. Imagine the student/
    trainee will be looking at the specimen/sample and needs to answer these questions. Use proper
    punctuation.

Return them individually as a JSON:

```
{
  "1": [{"question": str, "answer": str}, {"question": str, "answer": str}],
  "2": [{"question": str, "answer": str}, {"question": str, "answer": str}],
  etc.
}
```
````

Complementary question-answer pair generation prompt:

````
You are given a pathology report and a series of the questions. Answer the questions with just "Yes." or "No."
     based on the report.

Output your results in JSON format with the original questions and group every 2 questions together:

```json
{
    "1": [{"question": str, "answer": str}, {"question": str, "answer": str}],
    etc.
}
```
````

Open-ended question-answer pair generation prompt:

```
You are a pathology instructor at a world class medical school. Create one open-ended question/answer pair for
     each line in a given report, where answer is provided by the given line. The question should not be
    answerable with simply yes/no. Re-word the answer to introduce diverse linguistic patterns. Imagine the
    student/trainee will be looking at the specimen/sample and needs to answer these questions with no prior
    knowledge of the given report. Never use the word "noted". Use proper punctuation.

Return them individually as a JSON:

```
{
  "1": {"question": str, "answer": str},
  "2": {"question": str, "answer": str},
  etc.
}
```
```

Multiple-choice question-answer pair generation prompt:

```
Given a report from routine diagnostic examination of pathology WSIs for a case, create all the multiple
    choice questions that follow CAP protocols as closely as possible. Exclude measurements, laterality,
    lymph node counts, and IHC/molecular/cytology information. Do not make unnecessary reference to the
    history or specimen sections.

Also classify the template that you think best matches the given report according to these categories:
- Breast DCIS, Resection
- Breast DCIS, Biopsy
- Breast Phyllodes Tumor
- Breast Invasive, Resection
- Breast Invasive, Biopsy
- Central Nervous System
- Adrenal Gland
- Appendix NET
- Colon NET
- Duodenum and Ampulla NET
- Jejunum and Ileum NET
- Pancreas (Endocrine)
- Paraganglioma Pheochromocytoma
- Pituitary Neuroendocrine (PitNET)
- Stomach NET
- Thyroid
- Ampulla of Vater
- Anus, Excision
- Anus, Resection
- Appendix
- Colon and Rectum, Biopsy
- Colon and Rectum, Resection
- Distal Extrahepatic Bile Ducts
- Esophagus
- Gallbladder
- GIST, Biopsy
- GIST, Resection
- Hepatocellular Carcinoma
- Intrahepatic Bile Ducts
- Pancreas (Exocrine)
- Perihilar Bile Ducts
- Small Intestine
- Stomach
- Kidney, Biopsy
- Kidney, Resection
- Penis
- Prostate Needle Biopsy  Case Level
- Prostate Needle Biopsy  Specimen Level
```

```
- Prostate, Resection
- Prostate TURP
- Testis Orchiectomy
- Testis Lymphadenectomy
- Ureter, Renal Pelvis, Biopsy
- Ureter, Renal Pelvis, Resection
- Urethra, Biopsy
- Urethra, Resection
- Urinary Bladder, Resection
- Urinary Bladder, Biopsy
- Endometrium Uterus
- Ovary, Fallopian Tube, or Peritoneum
- Trophoblastic Tumors
- Uterine Cervix, Excision
- Uterine Cervix, Resection
- Uterine Sarcoma
- Vagina, Biopsy
- Vagina, Resection
- Vulva
- Larynx
- Oral Cavity
- Major Salivary Glands
- Nasal Cavity and Paranasal Sinuses
- Pharynx
- Cutaneous Squamous Cell Carcinoma
- Precursor and Mature Lymphoid Malignancies
- Myeloid and Mixed / Ambiguous Lineage Neoplasms
- Plasma Cell Malignancies
- Retinoblastoma
- Uveal Melanoma
- Ewing, Resection
- Ewing, Biopsy
- Germ Cell Tumor, Resection
- Germ Cell Tumor, Biopsy
- Hepatoblastoma, Resection
- Hepatoblastoma, Biopsy
- Neuroblastoma, Resection
- Neuroblastoma, Biopsy
- Rhabdomyosarcoma, Resection
- Rhabdomyosarcoma, Biopsy
- Wilms, Resection
- Wilms, Biopsy
- Invasive Melanoma of the Skin: Biopsy
- Invasive Melanoma of the Skin: Excision, Re-Excision
- Merkel Cell Carcinoma
- Lung, Resection
- Diffuse Pleural Mesothelioma
- Thymus
- Bone, Biopsy
- Bone, Resection
- Soft Tissue, Biopsy
- Soft Tissue, Resection

If there is not a matching protocol (i.e. because the sample is benign) then classify as "N/A".

Output your response in a JSON format as shown below:

Example:

user: Endometrioid carcinoma with squamous differentiation is present.

you:
{"cap_protocol": "Endometrium Uterus",
```

```
"questions": [{"question": "What is the histologic type?",
  "options": [
    "Endometrioid carcinoma, NOS",
    "Endometrioid carcinoma with squamous differentiation",
    "Endometrioid carcinoma, villoglandular variant",
    "Endometrioid carcinoma with secretory differentiation",
    "Endometrioid carcinoma, other variant (specify): ________________",
    "Serous endometrial intraepithelial carcinoma",
    "Serous carcinoma",
    "Carcinosarcoma (malignant mixed M\"ullerian tumor)",
    "Mucinous carcinoma",
    "Clear cell carcinoma",
    "Small cell neuroendocrine carcinoma",
    "Large cell neuroendocrine carcinoma",
    "Mixed cell carcinoma (specify types and percentages): __________________________",
    "Undifferentiated carcinoma",
    "Dedifferentiated carcinoma"
  ],
  "answer": "Endometrioid carcinoma with squamous differentiation"},
  etc.
  ]
}

Make sure that the answer matches one of the options.
```

S.6 Efficient Implementation

Sequence packing. Training efficiently on WSIs at scale presents several challenges for maximizing hardware utilization. The number of tile embeddings extracted from WSIs can vary dramatically between specimens, introducing highly variable sequence lengths within a training batch. A typical way to handle this is by padding to the longest sequence length in the batch, but this is not optimal as it introduces unnecessary compute and memory for the padding elements. Empirically, we find the ratio of compute/memory for padding elements to non-padding elements quickly exceeds 1.0 even with small batch sizes, which means we begin to dedicate the majority of compute/memory to padding elements. To mitigate this we implement sequence packing for the slide encoder which eliminates any need for padding and thus incurs no unnecessary computational and memory cost.

Dynamic batching. In multi-GPU data parallel training, variable length sequences also create load imbalance issues. For one training step, each data parallel worker (i.e. a GPU) does a forward and backward pass on its own mini-batch and then hits a global synchronization point for gradient `allreduce`. This synchronization point requires each worker to wait for the slowest worker to complete its training step, resulting in significant idle GPU time. Without careful batching, one GPU might process significantly more embeddings than another, creating severe load imbalance. Our sequence packing implementation directly enables efficient dynamic batching: we set a fixed budget of 800 thousand tile embeddings per GPU per step, then greedily pack variable-length sequences from a queue to fill this budget. This ensures each worker has the same amount of embeddings per step which improves workload balancing and mitigates idle GPU time. The number of specimens/reports per worker per step then becomes dynamic.

Efficient grouped query attention. We opt to use grouped query attention (GQA) [47] when doing cross attention with tile embeddings. This leads to significant memory savings during training by projecting tile embeddings to a much smaller embedding space as keys/values for cross attention. We use the official FlashAttention [57, 58] kernel from the `flash-attn` library which has native support for GQA rather than a naïve implementation that repeats key-value heads to match query heads which would negate the potential memory savings. The `flash-attn` kernel also supports sequence packing which allows us to avoid padding/unpadding before and after attention.

Fully sharded data parallelism and activation checkpointing. We use fully sharded data parallelism (FSDP) to shard model weights, gradients, and optimizer states. This reduces redundant memory and enables increasing model parameters and/or batch sizes. We noticed that, due to the relatively small batch size of text tokens, training of the decoder is bottlenecked by FSDP communications. We enabled activation checkpointing which reduces memory and allows increasing the batch size while still overlapping compute and communications.

S.7 Complete ablation results

	TITAN	PRISM	A	B	C	D	PRISM2
Pancancer	0.931 (0.924, 0.937)	0.947 (0.942, 0.953)	0.946 (0.940, 0.951)	0.945 (0.940, 0.950)	0.952 (0.947, 0.957)	0.954 (0.948, 0.959)	**0.967** (0.962, 0.971)
Pancancer (rare)	0.913 (0.902, 0.924)	0.931 (0.921, 0.940)	0.935 (0.926, 0.944)	0.933 (0.923, 0.942)	0.943 (0.934, 0.951)	0.943 (0.935, 0.951)	**0.957** (0.950, 0.964)
Breast (mean)	0.923 (0.917, 0.929)	0.975 (0.972, 0.979)	0.974 (0.970, 0.977)	0.974 (0.971, 0.978)	0.972 (0.968, 0.976)	0.976 (0.973, 0.980)	**0.980** (0.977, 0.983)
TCGA BRCA	0.956 (0.939, 0.970)	0.952 (0.932, 0.969)	0.955 (0.934, 0.972)	0.951 (0.929, 0.970)	0.954 (0.933, 0.971)	0.954 (0.933, 0.972)	**0.956** (0.933, 0.975)
TCGA NSCLC	0.981 (0.972, 0.988)	0.976 (0.967, 0.984)	0.982 (0.974, 0.989)	0.977 (0.969, 0.984)	0.975 (0.966, 0.982)	0.975 (0.966, 0.983)	**0.983** (0.976, 0.989)
TCGA RCC	**0.992** (0.987, 0.995)	0.985 (0.979, 0.991)	0.991 (0.987, 0.994)	0.987 (0.981, 0.992)	0.988 (0.983, 0.992)	0.986 (0.981, 0.990)	0.987 (0.982, 0.992)
Diagnostic	0.949	0.961	0.964	0.961	0.964	0.965	**0.972**

Supplementary Table S7: Diagnostic task linear probe performance for ablations: AUC, with 95% confidence intervals, computed with bootstrapping. Largest value across models per task in bold.

	TITAN	PRISM	A	B	C	D	PRISM2
IDC	0.954 (0.944, 0.963)	0.988 (0.983, 0.992)	0.986 (0.980, 0.991)	0.985 (0.977, 0.990)	0.989 (0.985, 0.992)	0.990 (0.986, 0.994)	**0.992** (0.989, 0.995)
ILC	0.958 (0.936, 0.978)	0.990 (0.978, 0.997)	**0.991** (0.987, 0.995)	0.990 (0.984, 0.995)	0.974 (0.952, 0.990)	0.991 (0.986, 0.995)	0.989 (0.980, 0.995)
DCIS	0.937 (0.926, 0.947)	0.982 (0.977, 0.986)	0.980 (0.974, 0.985)	0.982 (0.977, 0.987)	0.981 (0.976, 0.986)	0.984 (0.978, 0.988)	**0.987** (0.982, 0.991)
LCIS	0.893 (0.870, 0.913)	0.971 (0.962, 0.979)	0.964 (0.952, 0.974)	**0.973** (0.966, 0.980)	0.968 (0.956, 0.978)	0.970 (0.959, 0.978)	0.972 (0.961, 0.981)
ADH	0.865 (0.836, 0.890)	0.944 (0.929, 0.958)	0.938 (0.922, 0.953)	0.939 (0.923, 0.953)	0.936 (0.919, 0.951)	0.942 (0.926, 0.956)	**0.952** (0.938, 0.965)
ALH	0.887 (0.862, 0.911)	0.960 (0.942, 0.976)	0.965 (0.949, 0.978)	0.961 (0.944, 0.975)	0.962 (0.946, 0.977)	0.965 (0.948, 0.978)	**0.974** (0.962, 0.984)
Benign	0.936 (0.925, 0.945)	0.984 (0.980, 0.989)	0.985 (0.981, 0.989)	0.984 (0.979, 0.988)	0.985 (0.980, 0.989)	0.985 (0.981, 0.990)	**0.988** (0.984, 0.992)
Breast (mean)	0.923 (0.917, 0.929)	0.975 (0.972, 0.979)	0.974 (0.970, 0.977)	0.974 (0.971, 0.978)	0.972 (0.968, 0.976)	0.976 (0.973, 0.980)	**0.980** (0.977, 0.983)

Supplementary Table S8: Linear probe performance for breast (diagnostic) tasks for ablations: AUC, with 95% confidence intervals, computed with bootstrapping. Largest value across models per task in bold.

	TITAN	PRISM	A	B	C	D	PRISM2
MSK Biomarkers							
Prostate AR	0.911 (0.887, 0.932)	0.844 (0.814, 0.876)	0.914 (0.887, 0.938)	0.905 (0.877, 0.929)	0.909 (0.884, 0.931)	**0.923** (0.901, 0.944)	0.920 (0.898, 0.941)
Ovarian FGA	0.859 (0.766, 0.941)	0.848 (0.767, 0.921)	0.829 (0.729, 0.920)	0.767 (0.642, 0.871)	0.815 (0.707, 0.907)	0.838 (0.735, 0.925)	**0.865** (0.782, 0.933)
Esophagogastric Her2	**0.849** (0.747, 0.932)	0.809 (0.706, 0.897)	0.806 (0.711, 0.887)	0.790 (0.697, 0.879)	0.775 (0.679, 0.862)	0.806 (0.701, 0.891)	0.822 (0.736, 0.901)
Colorectal MSI	0.921 (0.888, 0.952)	0.958 (0.934, 0.977)	0.953 (0.930, 0.973)	0.953 (0.929, 0.972)	0.953 (0.930, 0.970)	0.949 (0.923, 0.972)	**0.965** (0.949, 0.978)
Breast CDH1	0.897 (0.871, 0.921)	0.913 (0.887, 0.936)	0.903 (0.878, 0.927)	0.903 (0.876, 0.926)	0.909 (0.882, 0.933)	0.904 (0.875, 0.930)	**0.917** (0.889, 0.940)
Bladder FGFR	**0.909** (0.870, 0.942)	0.895 (0.860, 0.927)	0.889 (0.849, 0.923)	0.884 (0.846, 0.920)	0.893 (0.852, 0.927)	0.893 (0.854, 0.928)	0.902 (0.866, 0.933)
Endometrial PTEN	**0.881** (0.851, 0.912)	0.880 (0.849, 0.911)	0.875 (0.845, 0.906)	0.872 (0.839, 0.904)	0.874 (0.842, 0.903)	0.876 (0.843, 0.906)	0.878 (0.845, 0.909)
Lung EGFR	0.813 (0.784, 0.838)	0.810 (0.779, 0.836)	0.820 (0.792, 0.846)	0.809 (0.781, 0.836)	0.806 (0.778, 0.833)	0.810 (0.784, 0.837)	**0.828** (0.803, 0.855)
Melanoma BRAF	0.757 (0.700, 0.811)	0.735 (0.684, 0.793)	0.725 (0.663, 0.786)	0.692 (0.636, 0.749)	0.695 (0.635, 0.756)	0.732 (0.679, 0.791)	**0.762** (0.706, 0.818)
Breast PIK3CA	0.658 (0.625, 0.691)	**0.686** (0.655, 0.718)	0.670 (0.636, 0.703)	0.667 (0.634, 0.700)	0.672 (0.641, 0.704)	0.673 (0.640, 0.704)	0.681 (0.649, 0.711)
MSK biomarkers (mean)	0.845 (0.829, 0.859)	0.838 (0.821, 0.852)	0.839 (0.822, 0.855)	0.824 (0.805, 0.841)	0.830 (0.814, 0.846)	0.840 (0.823, 0.857)	**0.854** (0.839, 0.867)
TCGA Biomarkers							
Colorectal MSI	0.864 (0.823, 0.903)	**0.890** (0.847, 0.926)	0.834 (0.774, 0.885)	0.852 (0.800, 0.896)	0.856 (0.808, 0.901)	0.877 (0.828, 0.922)	0.832 (0.779, 0.882)
Breast CDH1	0.912 (0.881, 0.940)	0.922 (0.885, 0.951)	0.921 (0.890, 0.945)	0.917 (0.884, 0.942)	0.914 (0.881, 0.942)	0.921 (0.891, 0.948)	**0.933** (0.910, 0.954)
Bladder FGFR	**0.826** (0.761, 0.885)	0.767 (0.687, 0.838)	0.794 (0.723, 0.858)	0.766 (0.689, 0.832)	0.786 (0.716, 0.857)	0.782 (0.709, 0.849)	0.806 (0.739, 0.873)
Endometrial PTEN	0.874 (0.839, 0.904)	0.863 (0.827, 0.896)	0.871 (0.836, 0.904)	0.870 (0.837, 0.901)	0.863 (0.828, 0.898)	0.867 (0.834, 0.901)	**0.876** (0.842, 0.908)
Lung EGFR	**0.822** (0.763, 0.876)	0.797 (0.735, 0.848)	0.806 (0.747, 0.863)	0.796 (0.730, 0.852)	0.807 (0.741, 0.868)	0.798 (0.733, 0.854)	0.800 (0.725, 0.862)
Melanoma BRAF	0.494 (0.344, 0.632)	0.462 (0.326, 0.595)	0.498 (0.356, 0.637)	0.536 (0.400, 0.689)	0.528 (0.397, 0.674)	0.533 (0.394, 0.677)	**0.547** (0.409, 0.682)
Breast PIK3CA	0.671 (0.634, 0.705)	0.671 (0.633, 0.708)	0.682 (0.648, 0.716)	0.685 (0.648, 0.721)	0.690 (0.653, 0.724)	0.685 (0.650, 0.723)	**0.694** (0.660, 0.728)
TCGA biomarkers (mean)	0.781 (0.754, 0.805)	0.767 (0.739, 0.795)	0.772 (0.744, 0.799)	0.775 (0.747, 0.801)	0.778 (0.752, 0.804)	0.781 (0.754, 0.804)	**0.784** (0.756, 0.810)
Biomarkers	0.813	0.803	0.805	0.799	0.804	0.811	**0.819**

Supplementary Table S9: Biomarker task linear probe performance for ablations: AUC, with 95% confidence intervals, computed with bootstrapping. A summary biomarker performance value is computed for each model as the average of 'MSK biomarkers (mean)' and 'TCGA biomarkers (mean)'. Largest value across models per task in bold.

	TITAN	PRISM	A	B	C	D	PRISM2
CNS	0.772 (0.754, 0.791)	0.774 (0.751, 0.797)	0.844 (0.825, 0.861)	**0.847** (0.831, 0.863)	0.841 (0.823, 0.858)	0.844 (0.827, 0.861)	0.837 (0.819, 0.854)
RCC	0.831 (0.796, 0.866)	0.828 (0.790, 0.863)	0.819 (0.780, 0.856)	0.822 (0.785, 0.855)	0.828 (0.796, 0.862)	0.828 (0.793, 0.864)	**0.842** (0.806, 0.875)
SARC	0.739 (0.688, 0.791)	0.712 (0.667, 0.758)	0.813 (0.757, 0.858)	0.798 (0.751, 0.848)	0.803 (0.752, 0.855)	0.813 (0.760, 0.858)	**0.843** (0.803, 0.882)
ENDO	**0.825** (0.786, 0.865)	0.807 (0.760, 0.848)	0.822 (0.782, 0.860)	0.791 (0.748, 0.829)	0.784 (0.744, 0.828)	0.774 (0.728, 0.819)	0.820 (0.777, 0.859)
BRCA	**0.765** (0.702, 0.819)	0.657 (0.580, 0.727)	0.722 (0.662, 0.785)	0.677 (0.612, 0.737)	0.674 (0.611, 0.733)	0.748 (0.672, 0.808)	0.731 (0.659, 0.792)
BLCA	0.679 (0.625, 0.727)	0.680 (0.634, 0.725)	0.664 (0.608, 0.719)	0.663 (0.608, 0.716)	0.668 (0.610, 0.721)	**0.690** (0.638, 0.744)	0.666 (0.613, 0.714)
CRC	0.706 (0.630, 0.772)	0.666 (0.585, 0.741)	**0.710** (0.644, 0.772)	0.709 (0.634, 0.773)	0.684 (0.611, 0.745)	0.700 (0.634, 0.767)	0.678 (0.604, 0.748)
LBTC	0.663 (0.602, 0.728)	**0.714** (0.649, 0.776)	0.624 (0.549, 0.703)	0.576 (0.504, 0.643)	0.685 (0.616, 0.745)	0.625 (0.560, 0.695)	0.679 (0.607, 0.745)
PAAD	0.646 (0.574, 0.716)	**0.654** (0.581, 0.727)	0.634 (0.559, 0.714)	0.593 (0.523, 0.662)	0.566 (0.488, 0.650)	0.615 (0.539, 0.693)	0.596 (0.514, 0.667)
NSCLC	0.611 (0.565, 0.659)	0.589 (0.540, 0.633)	0.620 (0.578, 0.663)	0.598 (0.551, 0.646)	0.621 (0.575, 0.666)	0.608 (0.562, 0.655)	**0.667** (0.625, 0.707)
MEL	0.552 (0.492, 0.614)	0.638 (0.571, 0.707)	0.603 (0.534, 0.669)	0.640 (0.575, 0.699)	**0.673** (0.612, 0.731)	0.615 (0.548, 0.673)	0.575 (0.505, 0.644)
HNSC	0.607 (0.548, 0.663)	0.601 (0.546, 0.660)	**0.632** (0.572, 0.690)	0.595 (0.539, 0.650)	0.590 (0.538, 0.650)	0.605 (0.553, 0.661)	0.616 (0.562, 0.668)
GI	0.584 (0.532, 0.637)	0.615 (0.562, 0.666)	0.679 (0.632, 0.729)	0.634 (0.576, 0.689)	0.644 (0.590, 0.697)	0.636 (0.583, 0.689)	**0.716** (0.671, 0.764)
TCGA survival (mean)	0.691 (0.675, 0.706)	0.687 (0.672, 0.702)	0.707 (0.690, 0.722)	0.688 (0.673, 0.704)	0.697 (0.681, 0.712)	0.700 (0.684, 0.716)	**0.713** (0.698, 0.728)
Survival	0.691	0.687	0.707	0.688	0.697	0.700	**0.713**

Supplementary Table S10: Survival task linear probe (CoxPH) performance for ablations: C-Index, with 95% confidence intervals, computed with bootstrapping. Largest value across models per task in bold.

	TITAN	PRISM	A	B	C	D	PRISM2
Pancancer	0.728 (0.717, 0.739)	0.793 (0.783, 0.802)	0.500 (0.500, 0.501)	0.716 (0.705, 0.727)	0.835 (0.825, 0.844)	0.860 (0.852, 0.869)	**0.876** (0.868, 0.884)
Pancancer (rare)	0.704 (0.686, 0.720)	0.753 (0.736, 0.770)	0.500 (0.500, 0.500)	0.697 (0.681, 0.712)	0.838 (0.825, 0.851)	0.834 (0.820, 0.847)	**0.857** (0.843, 0.870)
TCGA BRCA	0.895 (0.871, 0.916)	0.888 (0.866, 0.910)	0.491 (0.486, 0.495)	0.503 (0.497, 0.511)	0.775 (0.742, 0.809)	0.887 (0.860, 0.913)	**0.895** (0.866, 0.924)
TCGA NSCLC	**0.930** (0.914, 0.946)	0.907 (0.889, 0.924)	0.500 (0.500, 0.500)	0.694 (0.670, 0.717)	0.908 (0.890, 0.925)	0.907 (0.889, 0.924)	0.922 (0.906, 0.938)
PBI	0.814	0.835	0.498	0.653	0.839	0.872	**0.888**
TCGA RCC	0.900 (0.881, 0.916)	0.631 (0.597, 0.664)	0.335 (0.331, 0.340)	0.398 (0.373, 0.423)	0.401 (0.378, 0.423)	0.533 (0.505, 0.563)	**0.911** (0.885, 0.934)

Supplementary Table S11: Direct prediction performance for ablations: balanced accuracy, with 95% confidence intervals, computed with bootstrapping. A summary prompt-based inference (PBI) performance value is computed for each model as the average of Pancancer, Pancancer (rare), TCGA BRCA, and TCGA NSCLC. TCGA RCC is shown here but not considered in the average because the training data subset used for the ablation study lacks kidney samples and therefore, all kidney question-answer pairs are completely out-of-distribution for ablation models A, B, C, and D. Largest value across models per task in bold. Note that TITAN and PRISM use the contrastive approach and all ablations and PRISM2 are evaluated using question answering.

S.8 Tabulated linear probing results

This section presents all linear probing results from Fig. 4, 6, and 8 with the addition of linear probing on H-Optimus-0 slide-/block-/specimen-/patient-level embeddings created by taking the mean over all tile embeddings (similar to the Virchow2 Mean).

	Pan-cancer	Pan-cancer (rare)	Breast (Mean)	GI (Mean)
PRISM2 Diagnostic	**0.967** (0.962, 0.971)	**0.957** (0.950, 0.964)	**0.980** (0.977, 0.983)	**0.977** (0.975, 0.980)
PRISM2 Base	0.956 (0.951, 0.961)	0.943 (0.935, 0.952)	0.978 (0.975, 0.981)	**0.978** (0.976, 0.980)
PRISM	0.947 (0.942, 0.953)	0.931 (0.921, 0.940)	0.975 (0.972, 0.979)	0.971 (0.968, 0.973)
TITAN	0.931 (0.924, 0.937)	0.913 (0.902, 0.924)	0.923 (0.917, 0.929)	0.965 (0.961, 0.968)
Virchow2 Mean	0.917 (0.910, 0.923)	0.921 (0.911, 0.931)	0.937 (0.931, 0.944)	0.963 (0.959, 0.966)
H-Optimus-0 Mean	0.889 (0.880, 0.897)	0.903 (0.892, 0.914)	0.864 (0.853, 0.875)	0.959 (0.955, 0.962)
COBRA	0.913 (0.905, 0.919)	0.912 (0.901, 0.923)	0.883 (0.874, 0.892)	0.955 (0.951, 0.958)
GigaPath	0.876 (0.867, 0.883)	0.896 (0.885, 0.907)	0.849 (0.838, 0.859)	0.950 (0.947, 0.954)

Supplementary Table S12: MSK benchmarking: linear probe results for cancer, precursor, and benign condition detection, AUC (95% confidence intervals computed with 1000 iterations of bootstrapping). For each task, the statistically best result is in bold ($p < 0.05$, 1000 iterations of permutation testing).

	TCGA BRCA	TCGA NSCLC	TCGA RCC	CAMELYON16
PRISM2 Diagnostic	**0.956** (0.933, 0.975)	**0.983** (0.976, 0.989)	0.987 (0.982, 0.992)	**0.961** (0.905, 1.000)
PRISM2 Base	**0.964** (0.947, 0.979)	0.977 (0.968, 0.986)	**0.990** (0.986, 0.994)	**0.964** (0.914, 1.000)
PRISM	**0.952** (0.932, 0.969)	**0.976** (0.967, 0.984)	0.985 (0.979, 0.991)	**0.989** (0.970, 0.999)
TITAN	**0.956** (0.939, 0.970)	**0.981** (0.972, 0.988)	**0.992** (0.987, 0.995)	**0.979** (0.936, 1.000)
Virchow2 Mean	0.940 (0.920, 0.959)	0.972 (0.963, 0.981)	**0.992** (0.988, 0.996)	0.570 (0.451, 0.688)
H-Optimus-0 Mean	0.950 (0.934, 0.964)	0.975 (0.966, 0.983)	**0.991** (0.986, 0.995)	0.584 (0.473, 0.687)
COBRA	0.951 (0.932, 0.968)	0.977 (0.967, 0.984)	0.990 (0.985, 0.994)	0.625 (0.510, 0.730)
GigaPath	0.939 (0.921, 0.956)	0.964 (0.954, 0.975)	0.960 (0.949, 0.970)	0.617 (0.514, 0.715)

Supplementary Table S13: Public data (non-MSK) benchmarking: linear probe results for cancer subtyping or detection, AUC (95% confidence intervals computed with 1000 iterations of bootstrapping). For each task, the statistically best result is in bold ($p < 0.05$, 1000 iterations of permutation testing).

	CAMELYON17	PANDA-SI	PANDA-MI	IMP-CRS
PRISM2 Diagnostic	**0.881** (0.828, 0.925)	**0.862** (0.855, 0.869)	**0.930** (0.917, 0.943)	**0.952** (0.934, 0.967)
PRISM2 Base	**0.888** (0.837, 0.927)	0.836 (0.828, 0.844)	**0.921** (0.906, 0.936)	**0.950** (0.932, 0.967)
PRISM	0.852 (0.798, 0.890)	0.841 (0.833, 0.848)	0.911 (0.894, 0.926)	**0.944** (0.926, 0.960)
TITAN	0.641 (0.515, 0.752)	0.750 (0.738, 0.759)	0.901 (0.887, 0.914)	**0.944** (0.926, 0.960)
Virchow2 Mean	0.220 (0.029, 0.414)	0.279 (0.261, 0.296)	0.898 (0.879, 0.916)	0.933 (0.914, 0.950)
H-Optimus-0 Mean	0.049 (-0.107, 0.229)	0.332 (0.315, 0.349)	0.897 (0.877, 0.916)	0.914 (0.890, 0.935)
COBRA	0.020 (-0.021, 0.070)	0.613 (0.597, 0.629)	0.888 (0.867, 0.907)	0.907 (0.885, 0.927)
GigaPath	0.020 (0.000, 0.072)	0.628 (0.613, 0.644)	0.853 (0.829, 0.877)	0.917 (0.895, 0.936)

Supplementary Table S14: Public data (non-MSK) benchmarking: linear probe results for tumor grading and staging, quadratic weighted Kappa (95% confidence intervals computed with 1000 iterations of bootstrapping). For each task, the statistically best result is in bold ($p < 0.05$, 1000 iterations of permutation testing).

	IDC	ILC	DCIS	LCIS
PRISM2 Diagnostic	**0.992** (0.989, 0.995)	**0.989** (0.980, 0.995)	**0.987** (0.982, 0.991)	**0.972** (0.961, 0.981)
PRISM2 Base	0.988 (0.983, 0.993)	**0.986** (0.977, 0.992)	0.984 (0.980, 0.988)	**0.971** (0.961, 0.980)
PRISM	0.988 (0.983, 0.992)	**0.990** (0.978, 0.997)	0.982 (0.977, 0.986)	**0.971** (0.962, 0.979)
TITAN	0.954 (0.944, 0.963)	0.958 (0.936, 0.978)	0.937 (0.926, 0.947)	0.893 (0.870, 0.913)
Virchow2 Mean	0.953 (0.944, 0.962)	0.877 (0.832, 0.916)	0.944 (0.936, 0.953)	0.903 (0.883, 0.921)
H-Optimus-0 Mean	0.899 (0.880, 0.919)	0.785 (0.719, 0.843)	0.888 (0.867, 0.910)	0.826 (0.792, 0.858)
COBRA	0.931 (0.919, 0.944)	0.859 (0.802, 0.907)	0.901 (0.887, 0.914)	0.844 (0.816, 0.870)
GigaPath	0.896 (0.879, 0.911)	0.800 (0.748, 0.851)	0.847 (0.831, 0.864)	0.812 (0.783, 0.840)

	ADH	ALH	Benign	Mean
PRISM2 Diagnostic	**0.952** (0.938, 0.965)	**0.974** (0.962, 0.984)	**0.988** (0.984, 0.992)	**0.980** (0.977, 0.983)
PRISM2 Base	**0.951** (0.937, 0.962)	**0.968** (0.951, 0.981)	**0.987** (0.983, 0.991)	**0.978** (0.975, 0.981)
PRISM	**0.944** (0.929, 0.958)	0.960 (0.942, 0.976)	0.984 (0.980, 0.989)	0.975 (0.972, 0.979)
TITAN	0.865 (0.836, 0.890)	0.887 (0.862, 0.911)	0.936 (0.925, 0.945)	0.923 (0.917, 0.929)
Virchow2 Mean	0.921 (0.903, 0.937)	0.957 (0.943, 0.972)	**0.988** (0.983, 0.991)	0.937 (0.931, 0.944)
H-Optimus-0 Mean	0.847 (0.814, 0.878)	0.877 (0.848, 0.906)	0.891 (0.876, 0.905)	0.864 (0.853, 0.875)
COBRA	0.844 (0.813, 0.871)	0.855 (0.825, 0.881)	0.907 (0.896, 0.918)	0.883 (0.874, 0.892)
GigaPath	0.822 (0.791, 0.851)	0.834 (0.802, 0.861)	0.871 (0.857, 0.884)	0.849 (0.838, 0.859)

Supplementary Table S15: MSK benchmarking: linear probe results for breast cancer, precursor, and benign condition detection, AUC (95% confidence intervals computed with 1000 iterations of bootstrapping). For each task, the statistically best result is in bold ($p < 0.05$, 1000 iterations of permutation testing).

	Adenocarcinoma	Metastatic Tumor	Neuroendocrine Tumor	Lymphoma
PRISM2 Diagnostic	**0.995** (0.992, 0.997)	**0.986** (0.974, 0.994)	**0.983** (0.970, 0.992)	**0.980** (0.969, 0.990)
PRISM2 Base	**0.995** (0.994, 0.997)	**0.984** (0.972, 0.993)	**0.985** (0.975, 0.993)	**0.977** (0.963, 0.989)
PRISM	0.994 (0.991, 0.996)	0.978 (0.964, 0.990)	**0.980** (0.967, 0.991)	0.969 (0.952, 0.982)
TITAN	0.993 (0.991, 0.995)	0.971 (0.954, 0.986)	**0.977** (0.961, 0.990)	0.970 (0.955, 0.982)
Virchow2 Mean	0.986 (0.983, 0.989)	0.966 (0.949, 0.981)	0.953 (0.935, 0.969)	0.970 (0.956, 0.983)
H-Optimus-0 Mean	0.984 (0.981, 0.988)	0.955 (0.932, 0.973)	0.945 (0.925, 0.963)	0.964 (0.949, 0.978)
COBRA	0.981 (0.977, 0.985)	0.959 (0.940, 0.975)	0.934 (0.914, 0.955)	0.952 (0.938, 0.966)
GigaPath	0.979 (0.975, 0.982)	0.938 (0.913, 0.959)	0.920 (0.895, 0.941)	0.953 (0.934, 0.969)
	Squamous Cell Carcinoma	Tumor Differentiation	Barretts Esophagus	Polyp
PRISM2 Diagnostic	**0.996** (0.993, 0.999)	**0.999** (0.998, 0.999)	**0.990** (0.988, 0.992)	**0.979** (0.976, 0.981)
PRISM2 Base	0.995 (0.989, 0.999)	**0.999** (0.998, 0.999)	**0.990** (0.987, 0.992)	**0.979** (0.976, 0.982)
PRISM	0.994 (0.986, 0.999)	0.998 (0.997, 0.998)	0.988 (0.985, 0.991)	0.967 (0.963, 0.970)
TITAN	**0.994** (0.985, 0.999)	0.997 (0.997, 0.998)	0.962 (0.955, 0.969)	0.964 (0.960, 0.967)
Virchow2 Mean	**0.996** (0.994, 0.998)	0.992 (0.989, 0.994)	0.965 (0.958, 0.971)	0.968 (0.964, 0.971)
H-Optimus-0 Mean	**0.994** (0.992, 0.997)	0.992 (0.989, 0.994)	0.965 (0.958, 0.971)	0.966 (0.963, 0.970)
COBRA	0.992 (0.986, 0.996)	0.988 (0.984, 0.991)	0.960 (0.954, 0.967)	0.965 (0.961, 0.969)
GigaPath	0.993 (0.990, 0.996)	0.988 (0.985, 0.991)	0.955 (0.948, 0.962)	0.962 (0.958, 0.966)
	Dysplasia	Helicobacter Pylori	Inflammatory	Normal
PRISM2 Diagnostic	**0.951** (0.942, 0.959)	0.964 (0.944, 0.982)	**0.958** (0.954, 0.962)	**0.948** (0.944, 0.951)
PRISM2 Base	**0.950** (0.941, 0.958)	**0.978** (0.964, 0.989)	0.957 (0.953, 0.961)	**0.948** (0.944, 0.952)
PRISM	0.930 (0.920, 0.940)	0.966 (0.949, 0.982)	0.945 (0.940, 0.950)	0.942 (0.938, 0.946)
TITAN	0.912 (0.900, 0.924)	0.962 (0.939, 0.980)	0.937 (0.931, 0.942)	0.937 (0.933, 0.941)
Virchow2 Mean	0.917 (0.905, 0.928)	0.964 (0.940, 0.983)	0.939 (0.933, 0.944)	0.936 (0.932, 0.940)
H-Optimus-0 Mean	0.909 (0.897, 0.920)	0.966 (0.944, 0.982)	0.936 (0.931, 0.941)	0.930 (0.925, 0.934)
COBRA	0.906 (0.893, 0.917)	0.956 (0.930, 0.978)	0.934 (0.928, 0.939)	0.928 (0.924, 0.932)
GigaPath	0.898 (0.885, 0.910)	0.957 (0.933, 0.976)	0.929 (0.923, 0.934)	0.932 (0.928, 0.937)

Supplementary Table S16: MSK benchmarking: linear probe results for GI cancer, precursor, benign condition detection and cancer grading (tumor differentiation), AUC (95% confidence intervals computed with 1000 iterations of bootstrapping). For each task, the statistically best result is in bold ($p < 0.05$, 1000 iterations of permutation testing).

	Prostate AR	Ovarian FGA	Esophagogastric Her2	Colorectal MSI
PRISM2 Diagnostic	**0.915** (0.893, 0.935)	0.842 (0.750, 0.919)	**0.817** (0.732, 0.888)	0.949 (0.924, 0.970)
PRISM2 Base	**0.920** (0.898, 0.941)	**0.865** (0.782, 0.933)	**0.822** (0.736, 0.901)	**0.965** (0.949, 0.978)
PRISM	0.844 (0.814, 0.876)	0.848 (0.767, 0.921)	**0.809** (0.706, 0.897)	**0.958** (0.934, 0.977)
TITAN	**0.911** (0.887, 0.932)	0.859 (0.766, 0.941)	**0.849** (0.747, 0.932)	0.921 (0.888, 0.952)
Virchow2 Mean	0.889 (0.862, 0.916)	**0.894** (0.825, 0.950)	**0.818** (0.723, 0.901)	0.951 (0.930, 0.968)
H-Optimus-0 Mean	0.878 (0.850, 0.903)	**0.896** (0.824, 0.954)	**0.810** (0.719, 0.888)	0.952 (0.932, 0.970)
COBRA	0.882 (0.856, 0.908)	**0.919** (0.865, 0.961)	**0.816** (0.724, 0.900)	0.948 (0.926, 0.969)
GigaPath	0.876 (0.847, 0.904)	0.873 (0.795, 0.940)	**0.812** (0.714, 0.901)	0.936 (0.909, 0.959)
	Breast CDH1	Bladder FGFR	Endometrial PTEN	Lung EGFR
PRISM2 Diagnostic	**0.912** (0.885, 0.937)	**0.885** (0.846, 0.919)	**0.872** (0.841, 0.902)	0.816 (0.788, 0.842)
PRISM2 Base	**0.917** (0.889, 0.940)	**0.902** (0.866, 0.933)	**0.878** (0.845, 0.909)	**0.828** (0.803, 0.855)
PRISM	**0.913** (0.887, 0.936)	**0.895** (0.860, 0.927)	**0.880** (0.849, 0.911)	0.810 (0.779, 0.836)
TITAN	0.897 (0.871, 0.921)	**0.909** (0.870, 0.942)	**0.881** (0.851, 0.912)	0.813 (0.784, 0.838)
Virchow2 Mean	0.859 (0.824, 0.889)	**0.887** (0.845, 0.924)	**0.871** (0.838, 0.903)	**0.828** (0.800, 0.853)
H-Optimus-0 Mean	0.875 (0.842, 0.905)	0.872 (0.824, 0.915)	**0.865** (0.829, 0.894)	**0.837** (0.811, 0.863)
COBRA	0.866 (0.835, 0.895)	**0.887** (0.848, 0.920)	**0.871** (0.837, 0.901)	**0.839** (0.813, 0.863)
GigaPath	0.868 (0.832, 0.899)	0.881 (0.840, 0.917)	**0.859** (0.826, 0.892)	**0.823** (0.797, 0.850)
	Melanoma BRAF	Breast PIK3CA		
PRISM2 Diagnostic	**0.729** (0.668, 0.783)	**0.676** (0.644, 0.707)		
PRISM2 Base	**0.762** (0.706, 0.818)	**0.681** (0.649, 0.711)		
PRISM	**0.735** (0.684, 0.793)	**0.686** (0.655, 0.718)		
TITAN	**0.757** (0.700, 0.811)	0.658 (0.625, 0.691)		
Virchow2 Mean	0.739 (0.676, 0.795)	0.638 (0.603, 0.670)		
H-Optimus-0 Mean	0.735 (0.672, 0.792)	0.639 (0.605, 0.671)		
COBRA	**0.776** (0.717, 0.829)	0.654 (0.621, 0.687)		
GigaPath	**0.735** (0.670, 0.788)	0.618 (0.583, 0.652)		

Supplementary Table S17: MSK benchmarking: linear probe results for biomarker detection, AUC (95% confidence intervals computed with 1000 iterations of bootstrapping). For each task, the statistically best result is in bold ($p < 0.05$, 1000 iterations of permutation testing).

	Colorectal MSI	Breast CDH1	Bladder FGFR	Endometrial PTEN
PRISM2 Diagnostic	0.823 (0.759, 0.875)	**0.926** (0.897, 0.951)	0.757 (0.683, 0.832)	**0.875** (0.841, 0.906)
PRISM2 Base	0.832 (0.779, 0.882)	**0.933** (0.910, 0.954)	**0.806** (0.739, 0.873)	**0.876** (0.842, 0.908)
PRISM	**0.890** (0.847, 0.926)	**0.922** (0.885, 0.951)	0.767 (0.687, 0.838)	**0.863** (0.827, 0.896)
TITAN	**0.864** (0.823, 0.903)	0.912 (0.881, 0.940)	**0.826** (0.761, 0.885)	**0.874** (0.839, 0.904)
Virchow2 Mean	0.825 (0.765, 0.878)	0.868 (0.833, 0.900)	0.736 (0.651, 0.806)	0.832 (0.796, 0.869)
H-Optimus-0 Mean	0.848 (0.800, 0.896)	0.882 (0.849, 0.913)	0.720 (0.631, 0.797)	0.824 (0.785, 0.862)
COBRA	0.835 (0.771, 0.892)	0.909 (0.881, 0.933)	0.758 (0.677, 0.829)	0.838 (0.800, 0.873)
GigaPath	0.850 (0.802, 0.892)	0.853 (0.816, 0.886)	0.717 (0.626, 0.802)	0.803 (0.763, 0.839)
	Lung EGFR	Melanoma BRAF	Breast PIK3CA	
PRISM2 Diagnostic	0.784 (0.712, 0.843)	**0.495** (0.353, 0.629)	**0.694** (0.659, 0.728)	
PRISM2 Base	**0.800** (0.725, 0.862)	**0.547** (0.409, 0.682)	**0.694** (0.660, 0.728)	
PRISM	**0.797** (0.735, 0.848)	**0.462** (0.326, 0.595)	0.671 (0.633, 0.708)	
TITAN	**0.822** (0.763, 0.876)	**0.494** (0.344, 0.632)	**0.671** (0.634, 0.705)	
Virchow2 Mean	0.750 (0.672, 0.818)	0.432 (0.299, 0.575)	0.628 (0.588, 0.665)	
H-Optimus-0 Mean	0.756 (0.678, 0.821)	**0.449** (0.316, 0.584)	0.606 (0.574, 0.640)	
COBRA	**0.782** (0.708, 0.842)	**0.571** (0.424, 0.700)	0.664 (0.629, 0.698)	
GigaPath	0.761 (0.693, 0.827)	**0.467** (0.335, 0.607)	0.613 (0.576, 0.649)	

Supplementary Table S18: Public data (TCGA) benchmarking: linear probe results for biomarker detection, AUC (95% confidence intervals computed with 1000 iterations of bootstrapping). For each task, the statistically best result is in bold ($p < 0.05$, 1000 iterations of permutation testing).

	CNS	RCC	SARC	ENDO
PRISM2 Survival	**0.874** (0.858, 0.889)	**0.849** (0.815, 0.879)	**0.868** (0.825, 0.902)	**0.821** (0.774, 0.865)
PRISM2 Base	0.837 (0.819, 0.854)	**0.842** (0.806, 0.875)	**0.843** (0.803, 0.882)	**0.820** (0.777, 0.859)
PRISM2 Diagnostic	0.831 (0.814, 0.848)	**0.849** (0.816, 0.882)	0.819 (0.770, 0.865)	**0.830** (0.790, 0.869)
Survival Specialist	**0.863** (0.847, 0.880)	**0.837** (0.801, 0.875)	0.827 (0.780, 0.872)	**0.824** (0.777, 0.867)
PRISM	0.774 (0.751, 0.797)	**0.828** (0.790, 0.863)	0.712 (0.667, 0.758)	**0.807** (0.760, 0.848)
TITAN	0.772 (0.754, 0.791)	**0.831** (0.796, 0.866)	0.739 (0.688, 0.791)	**0.825** (0.786, 0.865)
Virchow2 Mean	0.844 (0.825, 0.861)	**0.823** (0.787, 0.857)	**0.843** (0.801, 0.883)	**0.804** (0.758, 0.851)
H-Optimus-0 Mean	0.845 (0.826, 0.862)	**0.821** (0.788, 0.855)	**0.864** (0.822, 0.901)	**0.787** (0.734, 0.838)
COBRA	0.824 (0.806, 0.844)	0.816 (0.783, 0.850)	0.821 (0.777, 0.864)	**0.799** (0.751, 0.841)
GigaPath	0.782 (0.764, 0.800)	0.802 (0.759, 0.842)	0.733 (0.681, 0.780)	0.781 (0.733, 0.827)

	BRCA	BLCA	CRC	LBTC
PRISM2 Survival	**0.799** (0.740, 0.852)	**0.736** (0.693, 0.780)	**0.744** (0.682, 0.805)	**0.692** (0.631, 0.750)
PRISM2 Base	**0.731** (0.659, 0.792)	0.666 (0.613, 0.714)	**0.678** (0.604, 0.748)	**0.679** (0.607, 0.745)
PRISM2 Diagnostic	0.719 (0.659, 0.773)	**0.673** (0.624, 0.723)	0.646 (0.570, 0.713)	**0.676** (0.611, 0.738)
Survival Specialist	**0.778** (0.716, 0.833)	**0.736** (0.691, 0.777)	**0.720** (0.652, 0.781)	**0.676** (0.608, 0.740)
PRISM	0.657 (0.580, 0.727)	**0.680** (0.634, 0.725)	**0.666** (0.585, 0.741)	**0.714** (0.649, 0.776)
TITAN	**0.765** (0.702, 0.819)	0.679 (0.625, 0.727)	**0.706** (0.630, 0.772)	**0.663** (0.602, 0.728)
Virchow2 Mean	**0.754** (0.686, 0.811)	**0.692** (0.646, 0.740)	**0.656** (0.578, 0.718)	**0.692** (0.622, 0.758)
H-Optimus-0 Mean	**0.773** (0.706, 0.831)	**0.676** (0.623, 0.729)	0.630 (0.549, 0.712)	**0.697** (0.632, 0.756)
COBRA	**0.749** (0.674, 0.811)	0.681 (0.626, 0.730)	**0.672** (0.601, 0.736)	**0.714** (0.646, 0.787)
GigaPath	**0.739** (0.673, 0.794)	0.598 (0.544, 0.653)	0.647 (0.569, 0.717)	**0.682** (0.608, 0.754)

	PAAD	NSCLC	MEL	HNSC
PRISM2 Survival	**0.662** (0.587, 0.736)	**0.674** (0.628, 0.715)	0.574 (0.504, 0.642)	**0.685** (0.631, 0.739)
PRISM2 Base	**0.596** (0.514, 0.667)	**0.667** (0.625, 0.707)	0.575 (0.505, 0.644)	0.616 (0.562, 0.668)
PRISM2 Diagnostic	0.550 (0.477, 0.623)	0.621 (0.575, 0.665)	**0.658** (0.596, 0.726)	0.610 (0.556, 0.668)
Survival Specialist	**0.672** (0.597, 0.738)	**0.670** (0.628, 0.713)	**0.667** (0.606, 0.730)	**0.655** (0.605, 0.707)
PRISM	**0.654** (0.581, 0.727)	0.589 (0.540, 0.633)	**0.638** (0.571, 0.707)	0.601 (0.546, 0.660)
TITAN	**0.646** (0.574, 0.716)	0.611 (0.565, 0.659)	0.552 (0.492, 0.614)	0.607 (0.548, 0.663)
Virchow2 Mean	**0.635** (0.562, 0.705)	**0.631** (0.583, 0.675)	**0.646** (0.583, 0.708)	0.612 (0.556, 0.670)
H-Optimus-0 Mean	**0.618** (0.542, 0.693)	0.558 (0.505, 0.604)	**0.665** (0.600, 0.724)	0.582 (0.521, 0.645)
COBRA	**0.590** (0.513, 0.662)	0.592 (0.542, 0.639)	**0.599** (0.492, 0.707)	0.551 (0.492, 0.609)
GigaPath	**0.589** (0.516, 0.668)	0.578 (0.529, 0.626)	**0.628** (0.568, 0.689)	0.603 (0.548, 0.661)

	GI	Mean
PRISM2 Survival	**0.692** (0.645, 0.743)	**0.744** (0.729, 0.758)
PRISM2 Base	**0.716** (0.671, 0.764)	0.713 (0.698, 0.728)
PRISM2 Diagnostic	**0.682** (0.629, 0.737)	0.705 (0.690, 0.719)
Survival Specialist	0.651 (0.593, 0.703)	**0.737** (0.723, 0.751)
PRISM	0.615 (0.562, 0.666)	0.687 (0.672, 0.702)
TITAN	0.584 (0.532, 0.637)	0.691 (0.675, 0.706)
Virchow2 Mean	0.650 (0.597, 0.701)	0.714 (0.699, 0.730)
H-Optimus-0 Mean	0.652 (0.595, 0.709)	0.705 (0.690, 0.720)
COBRA	0.657 (0.605, 0.709)	0.697 (0.680, 0.714)
GigaPath	0.640 (0.591, 0.692)	0.677 (0.660, 0.693)

Supplementary Table S19: Public data (TCGA) benchmarking: linear probe results for disease-specific survival (DSS) prediction, C-Index (95% confidence intervals computed with 1000 iterations of bootstrapping). For each task, the statistically best result is in bold ($p < 0.05$, 1000 iterations of permutation testing).

	CRC
PRISM2 Survival	**0.822** (0.767, 0.878)
PRISM2 Base	0.737 (0.669, 0.802)
PRISM2 Diagnostic	**0.765** (0.697, 0.831)
Survival Specialist	**0.784** (0.711, 0.855)
PRISM	0.743 (0.669, 0.811)
TITAN	0.724 (0.645, 0.792)
Virchow2 Mean	0.673 (0.587, 0.758)
H-Optimus-0 Mean	0.665 (0.583, 0.744)
COBRA	0.661 (0.577, 0.745)
GigaPath	0.589 (0.502, 0.672)

Supplementary Table S20: MSK benchmarking: linear probe results for recurrence-free survival (RFS) prediction in colorectal cancer, C-Index (95% confidence intervals computed with 1000 iterations of bootstrapping). For each task, the statistically best result is in bold ($p < 0.05$, 1000 iterations of permutation testing).

S.9 Training hyperparameters, architecture, and infrastructure

Hyperparameter	Stage 1	Stage 2
Base LR	1e-4	2e-5
Text Encoder LR	2e-5	N/A
Perceiver weight decay	0.1	N/A
Frozen modules	Phi-3-Mini	Perceiver
LR Schedule	Warmup → Constant	Warmup → Constant
Warmup Steps	500	500
Grad Accumulation Steps	5	5
Text token truncation	256	256
Max Tiles per GPU	800k	800k
Max Groups per GPU	30	30
Epochs	1	17
Caption Loss	1.0	1.0
Contrastive Loss	0.2	0.0
AdamW betas	0.9, 0.95	0.9, 0.95
Compute precision	bfloat16	bfloat16
Parallelism	FSDP	FSDP

Supplementary Table S21: Stage 1 and Stage 2 hyperparameters for model training.

Parameter	Value
Input Dimension	1280
Inner Dimension	2560
Latent Sequence Length	256
Cross-Attention KV Heads	10
Cross-Attention Q Heads	20
Latent Self-Attention Heads	20
Perceiver Depth	1
Self-Attention Depth	6
MLP Multiple	4
Activation	GELU
Pooler	Cross-Attention (256 → 1 latent)

Supplementary Table S22: Perceiver architecture details.

Parameter	Value
Type	MLP
Input Dimension	2560
Hidden Dimension	5120
Activation	GELU
Output Dimension	3072

Supplementary Table S23: Projector architecture details.

Parameter	Value
Compute Nodes	7
GPUs per Node	8
Total GPUs	56
GPU Architecture	A100
VRAM per GPU	40 GB
Min. inference requirements (base embedding)	1 × A10
Min. inference requirements (diagnostic embedding)	1 × A100

Supplementary Table S24: Compute infrastructure used for model training and inference.

S.10 Acronyms

**AMR**	adjusted mean recall
**AR**	androgen receptor
**AUC**	area under the curve
**BLN**	Breast Lymph Node
**BRAF**	B-Raf Proto-Oncogene
**CAP**	College of American Pathologists
**CDH1**	cadherin 1
**CRC**	colorectal cancer
**DCIS**	ductal carcinoma in situ
**dMMR**	deficient mismatch repair
**DSS**	disease-specific survival
**EGFR**	epidermal growth factor receptor
**FGA**	fraction of genome altered
**FGFR**	fibroblast growth factor receptor
**GI**	gastrointestinal
**HER2**	human epidermal growth factor receptor 2
**H&E**	hematoxylin and eosin
**IDC**	invasive ductal carcinoma
**IHC**	immunohistochemistry
**ILC**	invasive lobular carcinoma
**LLM**	large language model
**LOH**	loss-of-heterozygosity
**MLP**	multi-layer perceptron
**MMR**	mismatch repair

**mpp**	microns-per-pixel
**MSI**	microsatellite instability
**MSK-IMPACT**	MSK-Integrated Mutation Profiling of Actionable Targets
**MSK**	Memorial Sloan Kettering Cancer Center
**OS**	overall survival
**PTEN**	phosphatase and tensin homolog
**RFS**	recurrence-free survival
**TCGA**	The Cancer Genome Atlas
**WSI**	whole slide image
**QA**	question answering
