# Supplementary material for: End-to-end multimodal pathology foundation model with clinical dialogue
Source: Nat Med. 2026 Jul 31;32(9):3203–13. doi: 10.1038/s41591-026-04521-4 (PMC13577907; doi:10.1038/s41591-026-04521-4)
Supplement: Supplementary file 2 [file 41591_2026_4521_MOESM2_ESM.pdf]

nature portfolio

Corresponding author(s): Eugene Vorontsov

Last updated by author(s): May 22, 2026

Reporting Summary

Nature Portfolio wishes to improve the reproducibility of the work that we publish. This form provides structure for consistency and transparency in reporting. For further information on Nature Portfolio policies, see our Editorial Policies and the Editorial Policy Checklist.

Statistics

For all statistical analyses, confirm that the following items are present in the figure legend, table legend, main text, or Methods section.

n/a	Confirmed	
☐	☒	The exact sample size (*n*) for each experimental group/condition, given as a discrete number and unit of measurement
☒	☐	A statement on whether measurements were taken from distinct samples or whether the same sample was measured repeatedly
☐	☒	The statistical test(s) used AND whether they are one- or two-sided<br>*Only common tests should be described solely by name; describe more complex techniques in the Methods section.*
☒	☐	A description of all covariates tested
☒	☐	A description of any assumptions or corrections, such as tests of normality and adjustment for multiple comparisons
☐	☒	A full description of the statistical parameters including central tendency (e.g. means) or other basic estimates (e.g. regression coefficient) AND variation (e.g. standard deviation) or associated estimates of uncertainty (e.g. confidence intervals)
☐	☒	For null hypothesis testing, the test statistic (e.g. *F*, *t*, *r*) with confidence intervals, effect sizes, degrees of freedom and *P* value noted<br>*Give P values as exact values whenever suitable.*
☒	☐	For Bayesian analysis, information on the choice of priors and Markov chain Monte Carlo settings
☒	☐	For hierarchical and complex designs, identification of the appropriate level for tests and full reporting of outcomes
☒	☐	Estimates of effect sizes (e.g. Cohen's *d*, Pearson's *r*), indicating how they were calculated

Our web collection on statistics for biologists contains articles on many of the points above.

Software and code

Policy information about availability of computer code

Data collection	We used digitized histopathology images transferred from the Memorial Sloan Kettering Cancer Center and loaded with OpenSlide (v.1.3.1). Specimen- and report-level metadata were managed through internal Paige libraries. Dialogue generation and evaluation were implemented through the Paige internal reporting framework using the OpenAI API (openai v.0.28.1) with GPT4o model.
Data analysis	For statistical analysis, we used python (v.3.10) with scikit-learn (v.1.4.2) and pandas (v.2.2.2).

For manuscripts utilizing custom algorithms or software that are central to the research but not yet described in published literature, software must be made available to editors and reviewers. We strongly encourage code deposition in a community repository (e.g. GitHub). See the Nature Portfolio guidelines for submitting code & software for further information.

Data

Policy information about availability of data

All manuscripts must include a data availability statement. This statement should provide the following information, where applicable:

- Accession codes, unique identifiers, or web links for publicly available datasets
- A description of any restrictions on data availability
- For clinical datasets or third party data, please ensure that the statement adheres to our policy

This study did not specifically collect patient data. The retrospective analysis utilized proprietary deidentified digital pathology whole slides and associated metadata that were exclusively licensed by Paige.AI, Inc. from MSKCC. Requests for data need to be submitted to Paige AI (https://paige.ai/contact-us/) and evaluated by

Paige AI and MSKCC on a case-by-case basis. All requests complying with internal regulations on data privacy and intellectual property will be granted. All TCGA data used in this study are publicly available through the NCI Genomic Data Commons (GDC) portal at https://portal.gdc.cancer.gov/. The CAMELYON16 and CAMELYON17 datasets are publicly available via the Grand Challenge platform (https://camelyon16.grand-challenge.org/Data/ and https://camelyon17.grand-challenge.org/Data/). The PANDA challenge dataset is available via Kaggle at https://www.kaggle.com/c/prostate-cancer-grade-assessment/data. The IMP-CRS dataset is publicly accessible via the INESC TEC Research Data Management repository at https://rdm.inesctec.pt/dataset/nis-2023-008. To facilitate reproducibility, all pre-generated PRISM2 and baseline model embeddings, dataset tables, and linear probing evaluation code are hosted on Zenodo at https://zenodo.org/records/19056118.

Research involving human participants, their data, or biological material

Policy information about studies with human participants or human data. See also policy information about sex, gender (identity/presentation), and sexual orientation and race, ethnicity and racism.

Reporting on sex and gender	Gender or sex was not included as a covariate at any stage of our experimental analysis.
Reporting on race, ethnicity, or other socially relevant groupings	We did not use any socially constructed or socially relevant variables at any stage of this study.
Population characteristics	We did not collect or use any covariates pertaining to population characteristic at any stage of the study.
Recruitment	No patient recruitment was necessary for using histology whole slide images retrospectively.
Ethics oversight	Institutional review board review was not applicable for the research described in this study. This research study was conducted retrospectively from deidentified data licensed to Paige.AI, Inc. from Memorial Sloan Kettering (MSK). The data used in this study were all collected originally for clinical use by MSK in the practice setting and are therefore considered secondary data. Only data previously deidentified by MSK were utilized in the analysis, and unique patient identifiers were completely removed from the analytical dataset. To the best of our knowledge, MSK has not transferred any data for which the applicable patient has not consented to or otherwise agreed to MSK's Notice of Privacy Practices or a substantially similar notice, waiver or consent.

Note that full information on the approval of the study protocol must also be provided in the manuscript.

Field-specific reporting

Please select the one below that is the best fit for your research. If you are not sure, read the appropriate sections before making your selection.

☒ Life sciences ☐ Behavioural & social sciences ☐ Ecological, evolutionary & environmental sciences

For a reference copy of the document with all sections, see nature.com/documents/nr-reporting-summary-flat.pdf

Life sciences study design

All studies must disclose on these points even when the disclosure is negative.

Sample size	No sample size calculations were conducted.<br>A total of 685,507 part specimens and associated clinical reports (across 2,350,518 H&E whole slide images) were gathered for training the foundation model. The superior performance of our pretrained model compared to all other baselines indicates that the sample size was sufficient. For information on downstream datasets, please refer to the datasets and evaluation subsection in the Methods section of the manuscript.
Data exclusions	No particular data exclusion was performed.
Replication	Attempts at replication were successful for the reported model results. For further verification, the open-source model can be retrieved from https://huggingface.co/paige-ai/Prism2 and https://huggingface.co/paige-ai/Prism2-survival, along with linear probe code and all model embeddings (including baselines) for all public data at https://zenodo.org/records/19056118
Randomization	For downstream evaluation involving the creation of training, validation, and test splits, we utilized the official splits provided by the original investigators of each dataset wherever available. When such splits were not available, we created them randomly. Generally, we created random splits stratified by class, ensuring similar class proportions across splits, and, if possible, at the patient level, ensuring that slides from the same patient were kept in the same split. The random seeds were fixed, and the splits were documented to ensure replicability.
Blinding	Blinding is not necessary for our study.

Reporting for specific materials, systems and methods

We require information from authors about some types of materials, experimental systems and methods used in many studies. Here, indicate whether each material, system or method listed is relevant to your study. If you are not sure if a list item applies to your research, read the appropriate section before selecting a response.

Materials & experimental systems

n/a	Involved in the study
☒	☐ Antibodies
☒	☐ Eukaryotic cell lines
☒	☐ Palaeontology and archaeology
☒	☐ Animals and other organisms
☒	☐ Clinical data
☒	☐ Dual use research of concern
☒	☐ Plants

Methods

n/a	Involved in the study
☒	☐ ChIP-seq
☒	☐ Flow cytometry
☒	☐ MRI-based neuroimaging

Plants

Seed stocks	*Report on the source of all seed stocks or other plant material used. If applicable, state the seed stock centre and catalogue number. If plant specimens were collected from the field, describe the collection location, date and sampling procedures.*
Novel plant genotypes	*Describe the methods by which all novel plant genotypes were produced. This includes those generated by transgenic approaches, gene editing, chemical/radiation-based mutagenesis and hybridization. For transgenic lines, describe the transformation method, the number of independent lines analyzed and the generation upon which experiments were performed. For gene-edited lines, describe the editor used, the endogenous sequence targeted for editing, the targeting guide RNA sequence (if applicable) and how the editor was applied.*
Authentication	*Describe any authentication procedures for each seed stock used or novel genotype generated. Describe any experiments used to assess the effect of a mutation and, where applicable, how potential secondary effects (e.g. second site T-DNA insertions, mosiacism, off-target gene editing) were examined.*
